# Intermittent screening and treatment with artemisinin-combination therapy versus intermittent preventive treatment with sulphadoxine-pyrimethamine for malaria in pregnancy: a systematic review and individual participant data meta-analysis of randomised clinical trials

**DOI:** 10.1016/j.eclinm.2021.101160

**Published:** 2021-10-25

**Authors:** Julie R Gutman, Carole Khairallah, Kasia Stepniewska, Harry Tagbor, Mwayiwawo Madanitsa, Matthew Cairns, Anne Joan L'lanziva, Linda Kalilani, Kephas Otieno, Victor Mwapasa, Steve Meshnick, Simon Kariuki, Daniel Chandramohan, Meghna Desai, Steve M. Taylor, Brian Greenwood, Feiko O. ter Kuile

**Affiliations:** a Malaria Branch, Division of Parasitic Diseases and Malaria, Center for Global Health, Centers for Disease Control and Prevention, Atlanta, Georgia, USA; b Department of Clinical Sciences, Liverpool School of Tropical Medicine, Liverpool, UK; c WorldWide Antimalarial Resistance Network (WWARN), Oxford, UK; d Centre for Tropical Medicine and Global Health, Nuffield Department of Clinical Medicine, University of Oxford, Oxford, UK; e Infectious Diseases Data Observatory (IDDO), Oxford, UK; f University of Health and Allied Science, Ho, Ghana; g College of Medicine, University of Malawi, Blantyre, Malawi; h London School of Hygiene and Tropical Medicine, UK; i Kenya Medical Research Institute, Centre for Global Health Research, Kisumu, Kenya; j Department of Epidemiology, Gillings School of Global Public Health, University of North Carolina, Chapel Hill, NC, USA; k Division of Infectious Diseases and Duke Global Health Institute, Duke University Medical Center, Durham, NC, USA

**Keywords:** Malaria, pregnancy, intermittent preventive treatment, intermittent screening, sulphadoxine-pyrimethamine, artemisinin combination therapy

## Abstract

**Background:**

In sub-Saharan Africa, the efficacy of intermittent preventive therapy in pregnancy with sulphadoxine-pyrimethamine (IPTp-SP) for malaria in pregnancy is threatened by parasite resistance. We conducted an individual-participant data (IPD) meta-analysis to assess the efficacy of intermittent screening with malaria rapid diagnostic tests (RDTs) and treatment of RDT-positive women with artemisinin-based combination therapy (ISTp-ACT) compared to IPTp-SP, and understand the importance of subpatent infections.

**Methods:**

We searched MEDLINE and the Malaria-in-Pregnancy Library on May 6, 2021 for trials comparing ISTp-ACT and IPTp-SP. Generalised linear regression was used to compare adverse pregnancy outcomes (composite of small-for-gestational-age, low birthweight (LBW), or preterm delivery) and peripheral or placental *Plasmodium falciparum* at delivery. The effects of subpatent (PCR-positive, RDT/microscopy-negative) infections were assessed in both arms pooled using multi-variable fixed-effect models adjusting for the number of patent infections. PROSPERO registration: CRD42016043789.

**Findings:**

Five trials conducted between 2007 and 2014 contributed (10,821 pregnancies), two from high SP-resistance areas where *dhfr/dhps* quintuple mutant parasites are saturated, but sextuple mutants are still rare (Kenya and Malawi), and three from low-resistance areas (West-Africa). Four trials contributed IPD data (N=10,362). At delivery, the prevalence of any malaria infection (relative risk [RR]=1.08, 95% CI 1.00-1.16, I^2^=67.0 %) and patent infection (RR=1.02, 0.61-1.16, I^2^=0.0%) were similar. Subpatent infections were more common in ISTp recipients (RR=1.31, 1.05-1.62, I^2^=0.0%). There was no difference in adverse pregnancy outcome (RR=1.00, 0.96-1.05; studies=4, N=9,191, I^2^=54.5%). Subpatent infections were associated with LBW (adjusted RR=1.13, 1.07-1.19), lower mean birthweight (adjusted mean difference=32g, 15-49), and preterm delivery (aRR=1.35, 1.15-1.57).

**Interpretation:**

ISTp-ACT was not superior to IPTp-SP and may result in more subpatent infections than the existing IPTp-SP policy. Subpatent infections were associated with increased LBW and preterm delivery. More sensitive diagnostic tests are needed to detect and treat low-grade infections.

**Funding:**

Centers for Disease Control and Prevention and Worldwide Antimalarial Resistance Network.

**Research in context**

**Evidence before this study**

We conducted a literature search for trials in sub-Saharan Africa comparing intermittent screening and treatment in pregnancy (ISTp) with intermittent preventive treatment in pregnancy with sulfadoxine-pyrimethamine (IPTp-SP) in HIV-negative women using the search terms: ((intermittent AND screening) AND malaria) AND pregnan* AND Clinical Trial[ptyp] AND Humans[Mesh]; the following databases were searched: MEDLINE and the Malaria in Pregnancy Consortium (MiPc) Library, which consists of references from Web of Knowledge, Scopus, Cumulative Index to Nursing and Allied Health Literature (CINAHL), Bioline, the Cochrane Library databases, the World Health Organization (WHO) Global Health Library, as well as ‘grey literature’ and conference abstracts. The final search was conducted May 6, 2021. ISTp with artemisinin-based combination therapy was associated with a higher relative risk of malaria during pregnancy or at delivery and a non-significant increase in the risk of low birth weight. Modelling also suggested that screening with RDTs was less effective than IPTp-SP in the 2^nd^ and 3^rd^ trimester.

**Added value of this study**

This pooled analysis of five trials provides further evidence that ISTp with the current generation of rapid diagnostic tests (RDTs) is not superior to the existing strategy of IPTp with SP, regardless of the level of SP resistance. In addition, this is the largest individual patient data (IPD) meta-analysis of the impact of low-density infections below the level of detection by RDTs on clinically relevant pregnancy outcomes. The study provides evidence that these subpatent infections are potentially harmful and associated with poor birth outcomes, providing a possible explanation for the inferior efficacy of the ISTp strategies that rely on the current generation of RDTs.

**Implications of all the available evidence**

This study suggests that ISTp with the current generation of RDTs is not a suitable alternative to IPTp-SP even in high SP resistance areas where the dihydrofolate reductase/dihydropteroate synthase (dhfr/dhps) quintuple mutant parasites are saturated or have reached near saturation, but where the sextuple mutants are still rare, as is the case in most of East and southern Africa. Scale up of IPTp with SP in these areas should continue while simultaneously pursuing further research on alternative strategies, including assessing the use of more sensitive diagnostic tests to detect and treat more low-grade infections, either as ISTp or as an adjunct to IPTp-SP.

Alt-text: Unlabelled box

## 1 Introduction

Approximately 11.6 million pregnancies in sub-Saharan Africa were exposed to malaria in 2019 [1], a significant cause of poor birth outcomes.[2] In these areas, the World Health Organization (WHO) recommends that HIV-seronegative women receive intermittent preventive treatment in pregnancy (IPTp) with sulphadoxine-pyrimethamine (SP). Across east and southern Africa, resistance to SP is highly prevalent. The highest levels of resistance to SP undermine the ability of IPTp-SP to minimise the adverse consequences of malaria in pregnancy [3,4], necessitating alternative approaches [5,6].

One approach would be to use an alternative drug; however, poor tolerability undermines the effectiveness of amodiaquine, alone or combined with SP [7], mefloquine [8], and chloroquine either alone[9] or as chloroquine-azithromycin [10]. Dihydroartemisinin-piperaquine shows promise [11], [12], [13], and further trials with this combination are ongoing.

Another potential approach is to screen women with a rapid diagnostic test (RDT) at each scheduled antenatal care (ANC) visit and treat test-positive women with an effective drug. This approach is known as intermittent screening and treatment in pregnancy (ISTp). In two trials in West Africa, ISTp was non-inferior to IPTp-SP in reducing low birth weight (LBW) in areas with good parasite sensitivity to SP [14,15]. It was also well accepted by health care providers and pregnant women [16,17]. Two additional trials in East and southern Africa [11,18] indicated that screen-and-treat approaches were not superior to the existing strategy with IPTp-SP, despite being conducted in high resistance areas where the curative efficacy and duration of post-treatment prophylaxis provided by SP are reduced [3,19].

We carried out a systematic review and meta-analysis of individual-participant data (IPD) to comprehensively evaluate the potential utility of ISTp with a conventional RDT and an artemisinin combination therapy (ISTp-ACT) and define factors that determine the efficacy of ISTp-ACT relative to IPTp-SP in different settings. We also examined the effect of subpatent malaria infections missed by rapid diagnostic tests but detected by nucleic acid amplification tests like polymerase chain reaction (PCR) on adverse pregnancy outcomes in these trials.

## 2 Methods

### 2.1 Search strategy and selection criteria

An electronic literature search, using the search terms: ((intermittent AND screening) AND malaria) AND pregnan* AND Clinical Trial[ptyp] AND Humans[Mesh]) was conducted on August 8, 2016, and updated on May 6, 2021, following PRISMA guidelines [20]. The following databases were searched: MEDLINE and the Malaria in Pregnancy Consortium (MiPc) Library, which includes references from Web of Knowledge, Scopus, CINAHL, Bioline, the Cochrane Library databases, WHO Global Health Library, as well as ‘grey literature’ and conference abstracts [21]. A multi-concept Boolean search strategy was applied using keywords and MeSH terms. Randomised controlled trials among pregnant women comparing ISTp-ACT versus IPTp-SP were eligible (Supplement 1, page 2). The search was conducted in English but without language or date restriction.

### 2.2 Data extraction and quality assessment

Two independent reviewers (JRG and MM or CK) screened titles, abstracts, and full texts of all citations. For eligible studies, authors were contacted to request de-identified individual-level data. Three attempts were made to contact authors. Data were analysed using STATA/MP2 16.0 (StataCorp LP), according to an *a priori* defined statistical analysis plan. Reviewers were unblinded to the authors of the source study. Two reviewers (JRG and CK) independently assessed the risk of bias for the included trials using the Cochrane risk-of-bias tool for randomised trials version 2 (RoB2) [22] (Supplement 2, page 2). The study is registered in PROSPERO (CRD42016043789).

### 2.3 Outcomes

The co-primary outcomes for the comparison of the effect of ISTp-ACT vs IPTp-SP were 1) maternal malaria infection at delivery, defined as any *Plasmodium* infection detected in peripheral or placental blood by PCR, microscopy, RDT, or histopathology (acute and/or chronic infection) and 2) adverse live-birth, defined as the composite of LBW (<2500 grams), small-for-gestational-age (SGA, <10^th^ percentile relative to INTERGROWTH-21^st^ gender-specific chart) [23], or preterm delivery (<37 weeks gestation).

Secondary outcome definitions are provided in Supplement 3, page 2. The analyses of the impact of ISTp-ACT versus IPTp-SP during pregnancy excluded enrolment and delivery time points from incidence data. Analysis of the impact of patent and subpatent (PCR-positive, RDT/microscopy-negative) infection on adverse pregnancy outcomes included enrolment and delivery time points.

### 2.4 Statistics

#### 2.4.1 Efficacy analyses of ISTp vs IPTp

Log-binomial generalised linear regression models were used to obtain risk ratios (RR) and 95% confidence intervals (CI). When convergence was not achieved, we followed suggestions from Cummings.[24] For continuous outcomes, mean differences and 95% CIs were obtained from linear regression.

The primary analyses of the primary and secondary outcomes used two-stage IPD meta-analysis. Because the number of studies was small, fixed-effect models were used [25,26]. Because IPD data was not available for one study [27], aggregated data of this study were included in the second stage [28]. Unadjusted models were used for the primary analysis. The stratification factors study site (i.e., health facility) and gravidity were included *a priori* as covariates in all unadjusted models because the randomisation was stratified by study site in all IPD studies and by gravidity in Malawi [18]. Heterogeneity was measured using the *I*^2^ statistic [29] (Supplement 4, page 3). To explore potential modification of treatment effect by prevailing SP resistance levels, we classified studies into low (≤90% prevalence of *Plasmodium falciparum dihydropteroate synthase (Pfdhps)* K540E mutation) and high (>90% *Pfdhps*K540E) resistance [3].

Supportive secondary analyses using covariate adjusted and subgroup analyses were performed using the 2-stage model. Potential effect modifiers and confounding variables were pre-specified. In addition to the stratification factors study site and gravidity, these included the baseline factors maternal haemoglobin concentration, bed net use, and gestational age. Maternal socioeconomic status, maternal education, and malaria status at enrolment were excluded because they were missing in a large proportion of participants in at least one of the studies.

Further sensitivity analyses to assess the robustness of the primary analysis were conducted using 1-stage models, with site and gravidity included as covariates, and random-effect models.

#### 2.4.2 Analysis of the impact of subpatent malaria on pregnancy outcomes

The effect of exposure to subpatent malaria on pregnancy outcomes was examined using fixed-effect models with robust Poisson regression for binary outcomes and linear regression for continuous outcomes, accounting for study and the total number of malaria tests conducted, including the number of patent infections detected. In the binary models, risk ratios (RR) correspond to the change in the risk of the adverse outcome associated with one additional positive test (i.e. a patent or subpatent malaria infection) during pregnancy. In models with continuous outcomes, the mean difference in the outcome measure associated with each additional patent or subpatent infection was estimated. Both types of models included a robust estimator of variance. Crude models included study arm and the number of patent and subpatent infections as the two exposure variables of interest. Adjusted models (primary analysis) also included gestational age at enrolment, maternal age, gravidity (paucigravidae [G1-G2]/multigravidae), and the number of sick visits (Supplement 5, page 3). A sensitivity analysis was conducted to determine if the method used to assess gestational age influenced the conclusions for outcomes requiring gestational age at delivery (preterm delivery, SGA) (Supplement 6, page 4; Supplement 8, page 12; Table S5).

Details of the performance of the RDTs to detect PCR-positive infections have been described previously [30].

### 2.5 Ethics

The five original studies were approved by the relevant local and international partner ethical committees and institutional review boards. The protocol for the meta-analysis was reviewed by the US Centers for Disease Control and Prevention (CDC) Human Research Protection Office and deemed exempt from further review. Written consent was required from each patient for participation in each individual study.

### 2.6 Role of the funding sources

WWARN had no role in study design, data collection, data analysis, data interpretation, or writing of the report. CDC staff were involved in study design, data collection, data analysis, data interpretation, and writing of the report. The corresponding author had full access to all the data in the study and had final responsibility for the decision to submit for publication.

## 3 Results

### 3.1 Studies and baseline characteristics

**Figure 1:**
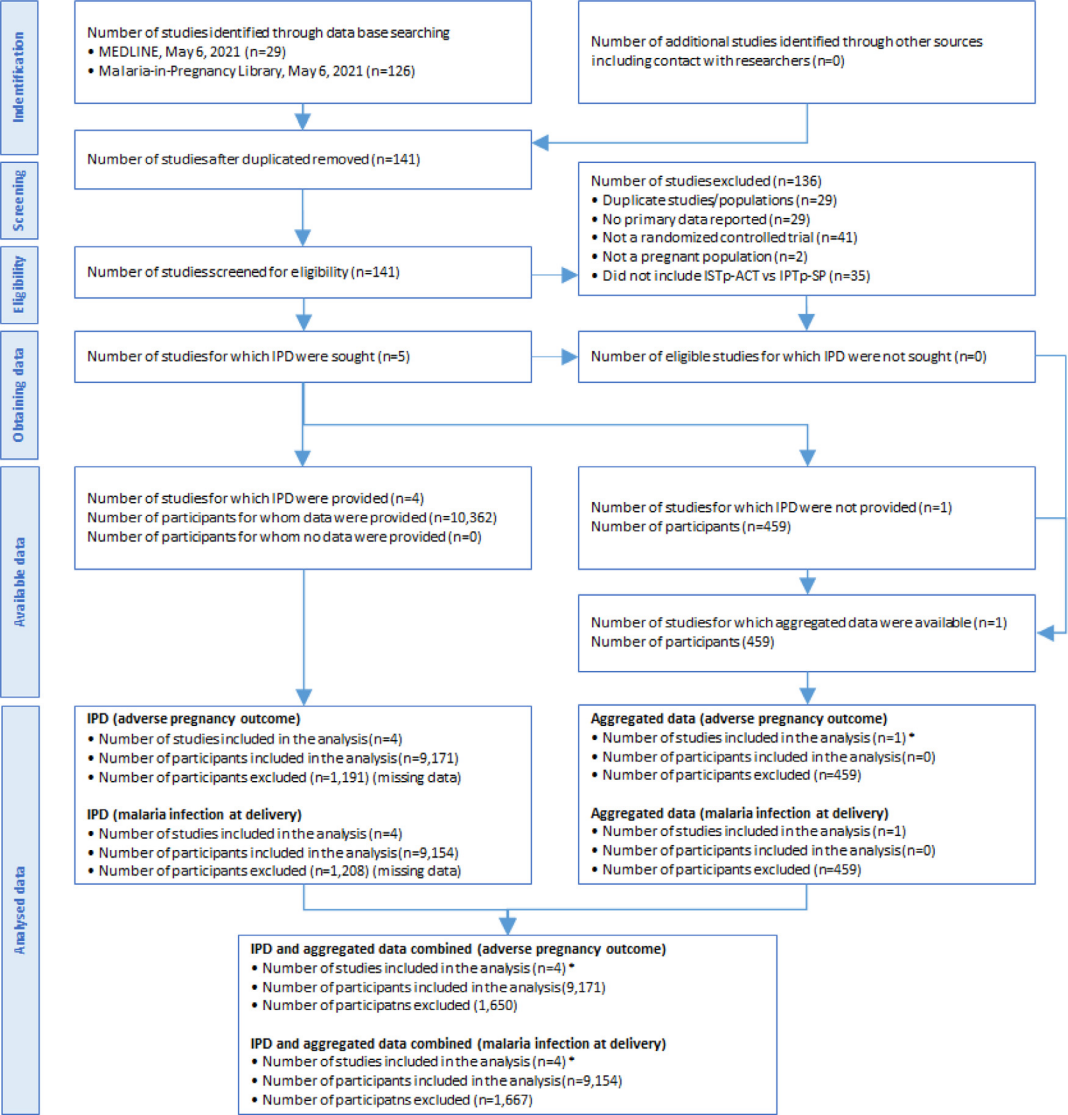
PRISMA diagram of included studies *For the study in Nigeria (Esu et al., 2018, n=459), data on the primary outcome used in the meta-analysis were not available. However, data on haemoglobin (n=236), anaemia (n=236), and malaria infection (n=208) in the third trimester, perinatal death (420), pregnancy outcome (n=418), preterm birth (n=420), mean birthweight and LBW (n=325) were available. IPD = individual patient data

A total of 141 studies were reviewed (Figure 1 and Supplement 7, page 4). Five trials met the eligibility criteria and were included in the analysis; one from Ghana (N=2,205 pregnancies) [14], one four-country trial in West Africa (N=5,292) [15], one from Malawi (N=1,844) [18], one from Kenya (N=1,021) [11], and one from Nigeria (N=459) [27] (Table S1). SP resistance was classified as low for three studies and high for two studies [11 18], None were in areas with “super resistance” characterized by >90% prevalence of *Pfdhps*K540E and >10% *Pfdhps*A581G [31]. For the first four trials, individual participant data were available (N=10,362 pregnancies) (Table S2). The results of the Nigerian trial [27] were added as aggregated data in the second stage for eight binary and two continuous outcomes. The first four trials were judged to have a low risk of bias. The Nigerian trial was judged to have a high risk of bias (Figure S1).

Among the 10,362 women included in the IPD analysis, the baseline characteristics were well balanced between the study arms (Table 1). The mean age was 22.4 years, the mean gestational age 20.7 weeks, 42.5% were primigravidae, and just over half reported using a bednet the night before enrolment. Of the 5187 women in the ISTp arm, 2254 (43.5%) had at least one positive RDT and were treated with an ACT. Overall a total of 3,053 ACT treatments were given to these 2,254 women (mean 1.3, median 1, range 1-4), whereas 13,247 IPTp courses with SP were given to 5,154 women in the IPTp arm (mean 2.6, median 2, range 1-7).

**Table 1:** Baseline characteristics by study arm

		ISTp	IPTp	Overall
		(n=5187)	(n=5175)	(N=10,362)
Maternal characteristics				
Maternal age (years)		22.4 (5.2)	22.4 (5.1)	22.4 (5.2)
Used a bednet previous night		2617 (54.2%)	2659 (55.0%)	5276 (54.6%)
Schooling level†				
	Low	2874 (59.1%)	2901 (60.0%)	5775 (59.5%)
	Medium	1629 (33.5%)	1563 (32.3%)	3192 (32.9%)
	High	362 (7.4%)	375 (7.7%)	737 (7.6%)
SES index score (median, range)		-0.1 (-8.3; 12.5)	-0.1 (-7.9; 19.6)	-0.1 (-8.3; 19.6)
Pregnancy number (gravidity)				
	First	2216 (42.8%)	2173 (42.1%)	4389 (42.5%)
	Second	1764 (34.1%)	1802 (34.9%)	3566 (34.5%)
	Third or higher	1194 (23.1%)	1184 (23.0%)	2378 (23.0%)
Gestational age (weeks)		20.7 (3.4)	20.7 (3.4)	20.7 (3.4)
Weight (kg)		58.0 (9.5)	58.5 (9.6)	58.2 (9.5)
Height (cm)		156.5 (7.9)	156.8 (7.9)	156.6 (7.9)
Laboratory findings				
Haemoglobin (g/dL)		10.5 (1.8)	10.6 (2.0)	10.6 (1.9)
Malaria infection				
	RDT‡	1616 (31.7%)	114 (48.1%)	1730 (32.4%)
	Microscopy	1208 (23.9%)	1213 (24.1%)	2421 (24.0%)
	PCR	1566 (40.2%)	768 (41.2%)	2334 (40.6%)
	Subpatent	493 (17.4%)	369 (12.5%)	862 (14.9%)

ISTp=intermittent screening and treatment in pregnancy. IPTp=intermittent preventive treatment in pregnancy.

*Values are mean (SD) or percentages unless otherwise indicated.

† Schooling: Low: no schooling or primary school not completed, Medium: Primary school completed, High: Junior high school completed, Highest: Senior High school or academy completed.

‡ The RDT data at enrolment between study arms are not comparable because of the nature of the intervention; RDT were only conducted in symptomatic women in the IPTp arms, but among all women in the ISTp arm

### 3.2 Primary efficacy outcomes

**Figure 2:**
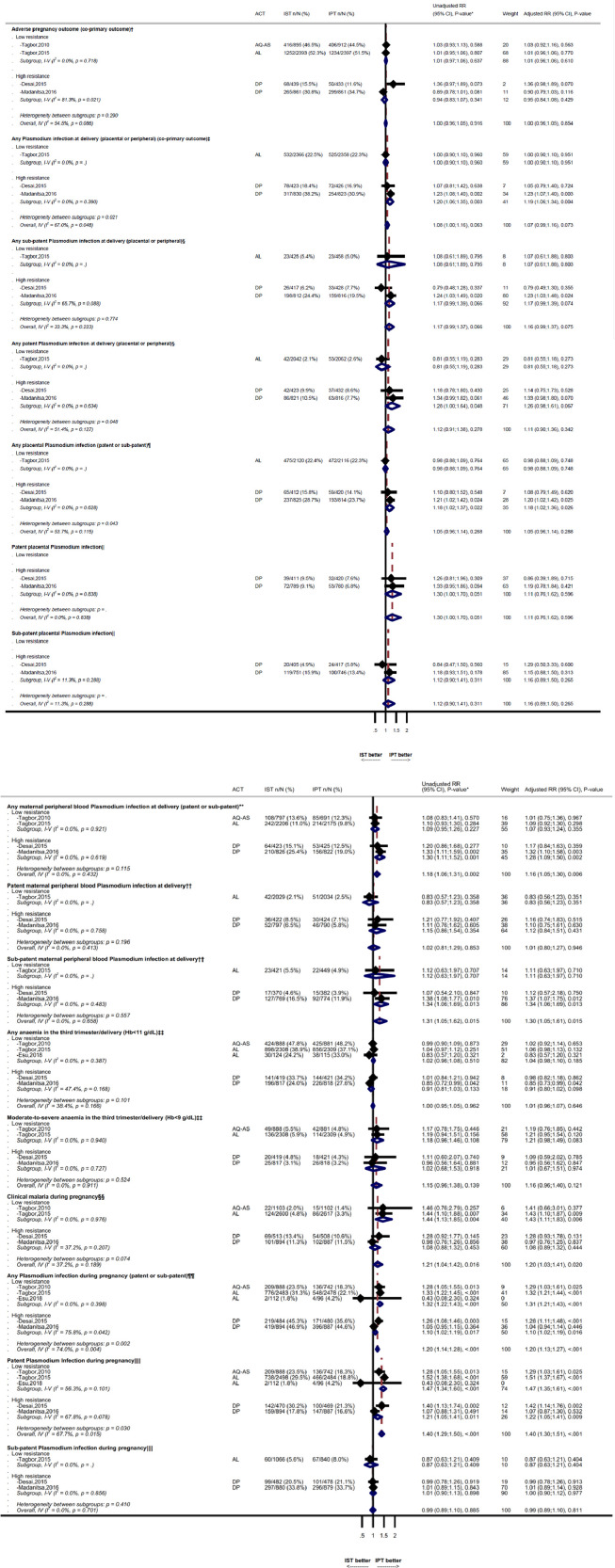
Co-primary outcomes (adverse pregnancy outcome, and malaria at delivery) and secondary maternal outcomes ACT=artemisinin-based combination therapy, AQ-AS= amodiaquine-artesunate, AL=Artemether-lumefantrine, DP=dihydroartemisinin-piperaquine, IST=Intermittent screening and treatment, IPT=intermittent preventive treatment, RR=relative risk, CI=confidence interval, PCR=polymerase chain reaction. The outcomes during pregnancy reflect the cumulative risk. Weight reflects fixed-effect models. *Unadjusted relative risk models include the stratification factors site and gravidity (primigravidae vs secundigravidae vs multigravidae) for all four studies for which IPD data was available, as these were used in some of the source studies. Adjusted models also include anaemia at enrolment (haemoglobin < 11 g/dL), gestational age (binary, study-specific median), and maternal ITN use at enrolment. ^†^Adverse live-birth (co-primary outcome) defined as the composite of low birth weight (<2500 grams), small-for-gestational-age (SGA, <10th percentile relative to INTERGROWTH-21st gender-specific chart), or and preterm delivery (<37 weeks gestation). ^‡^Any malaria at delivery (co-primary outcome) defined as any maternal plasmodium infection detected in peripheral or placental blood by any diagnostic method (PCR, microscopy, RDT or histopathology (acute and/or chronic infections)). ^§^Any patent *Plasmodium* infection in peripheral or placental blood detected by PCR or histopathology (acute and/or chronic infections) and positive by microscopy or RDT. Any subpatent infection at delivery is defined as a microscopy and RDT negative infection detected by PCR or histopathology. ^¶^Any placental malaria infection detected in the placental blood by any diagnostic method (PCR, microscopy, RDT or histopathology (acute and/or chronic infections)). ^||^Patent placental malaria infection defined as any infection in the placental blood detected by PCR or histopathology positive by RDT or microscopy. Subpatent placental malaria infection is defined as microscopy and RDT negative infections detected by PCR or histopathology (acute and/or chronic infections). ^⁎⁎^Any maternal plasmodium infection detected in peripheral blood by PCR, microscopy, or RDT. ^††^Patent maternal plasmodium infection in peripheral blood detected by PCR and by microscopy or RDT. Subpatent maternal plasmodium infection detected in peripheral blood by PCR, but not by microscopy or RDT ^‡‡^Maternal anaemia (Hb <11 g/dL) and moderate to severe anaemia (Hb <9 g/dL) at delivery or otherwise in the third trimester if values at delivery were not available. §§Clinical malaria, defined as documented fever or recent history of fever in the presence of microscopy or RDT confirmed malaria infection. ¶¶Any maternal peripheral blood *Plasmodium* infection during pregnancy, detected by microscopy or RDT, or PCR. ||||Patent maternal peripheral blood *Plasmodium* infection during pregnancy detected by PCR and by microscopy or RDT. Subpatent Plasmodium infection during pregnancy, defined as PCR positive but microscopy and RDT negative infections. The effect sizes for primary outcomes in a sensitivity analysis using 1-stage models were 1.00 (95% CI 0.96-1.05, p=0.9) for adverse pregnancy outcome and 1.07 (95% CI 1.00-1.15, p=0.06) for any malaria infection at delivery, respectively.

Data for the co-primary outcomes were available from the four studies with IPD but not from the Nigerian study [27]. Women in the ISTp arm were similarly likely at delivery to have peripheral or placental malaria detected by any measure (RDT/smear/PCR/histopathology) (RR=1.08, 95% confidence interval (CI) 1.00-1.16; p=0.06; I^2^=67.0%, N=7,226, N=3 trials, Figure 1). There was no difference in the composite adverse pregnancy outcomes (LBW/SGA/PTD) (RR=1.00, 0.96-1.05, p=0.92, I^2^=54.5%, N=9,191, N=4, Figure 2). Random-effects models produced similar results (Table S3).

### 3.3 Secondary efficacy outcomes

#### 3.3.1 Maternal outcomes

During pregnancy, women in the ISTp arm had a 21% increased risk of developing clinical malaria compared to the IPTp arm (RR=1.21, 95% CI 1.04-1.42, p=0.02, N=4 trials, I^2^=37.2%), and a 40% increased risk of patent infection (RR=1.40, 1.29-1.50, p<0.001, N=5, I^2^=67.7%), but not subpatent infection (RR=0.99, 0.89-1.10, p=0.89, N=4, I^2^=0%). At delivery, women in the ISTp arm had an 18% increased risk of any peripheral-blood malaria infection (RR=1.18, 1.06-1.31, p=0.002, I^2^=0%), reflecting 31% more subpatent infections (RR=1.31, 1.05-1.62, p=0.01, I^2^=0%), but not more patent infections (RR=1.02, 0.81-129, p=0.85, I^2^=0%) (Figure 2). There were no differences between study arms in placental malaria (RR=1.05, 0.96-1.14, p=0.27, I^2^=53.7%, N=3), maternal anaemia (haemoglobin <11 g/dL [p=0.97] and <9 g/dL [p=0.14]) (Figure 1), or mean haemoglobin (p=0.69) (Table S4).

#### 3.3.2 Neonatal outcomes

**Figure 3:**
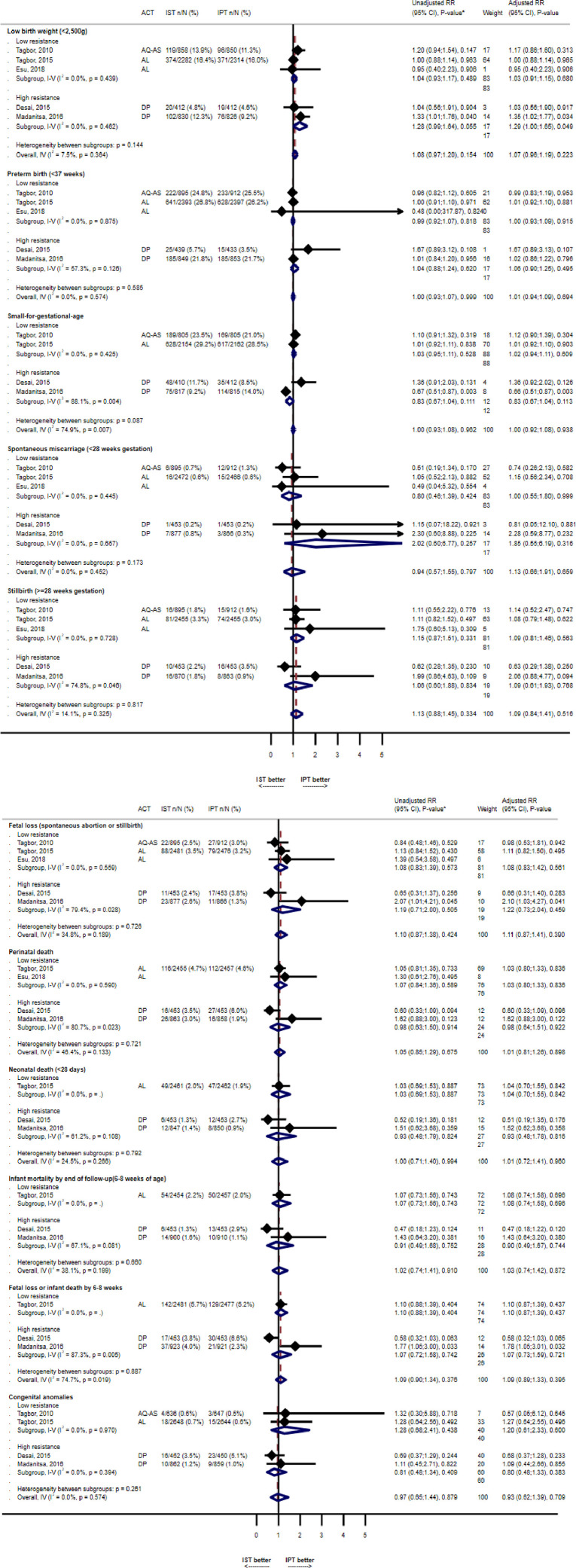
Neonatal outcomes comparing ISTp to IPTp *Unadjusted relative risk models include the stratification factors site and gravidity (primigravidae vs secundigravidae vs multigravidae) for all four studies for which IPD data was available, as these were used in some of the source studies. Adjusted models also include anaemia at enrolment (haemoglobin < 11 g/dL), gestational age (binary, study-specific median), and maternal ITN use at enrolment ACT=artemisinin-based combination therapy, AQ-AS= amodiaquine-artesunate, AL=Artemether-lumefantrine, DP=dihydroartemisinin-piperaquine, IST=Intermittent screening and treatment, IPT=intermittent preventive treatment, RR=relative risk, CI=confidence interval.

The risk of LBW infants was similar between arms (RR=1.08, 0.97-1.20, p=0.15, N=5, I^2^=7.5%), but the mean birthweight was 28g lower in IST recipients (95% CI 10-46, p=0.003, N=5, I^2^=0%). None of the other neonatal outcomes were significantly different between arms (Figure 3).

### 3.4 Impact of patent and subpatent infections on adverse pregnancy outcomes

Data on patent and subpatent infection were available from three of the five trials [11,15,18]. Overall, 2,352 (32.3%) of 7,283 women with PCR data had at least one patent *Plasmodium* infection during pregnancy (including enrolment and delivery), and 1,632 (22.4%) had at least one subpatent infection. About three-quarters (74.7%) of the 1,632 women with subpatent infections did not have evidence of any patent infection during pregnancy or delivery. Similarly, 29.8% of the 2,352 women with patent infections did not have additional subpatent infections identified.

**Figure 4:**
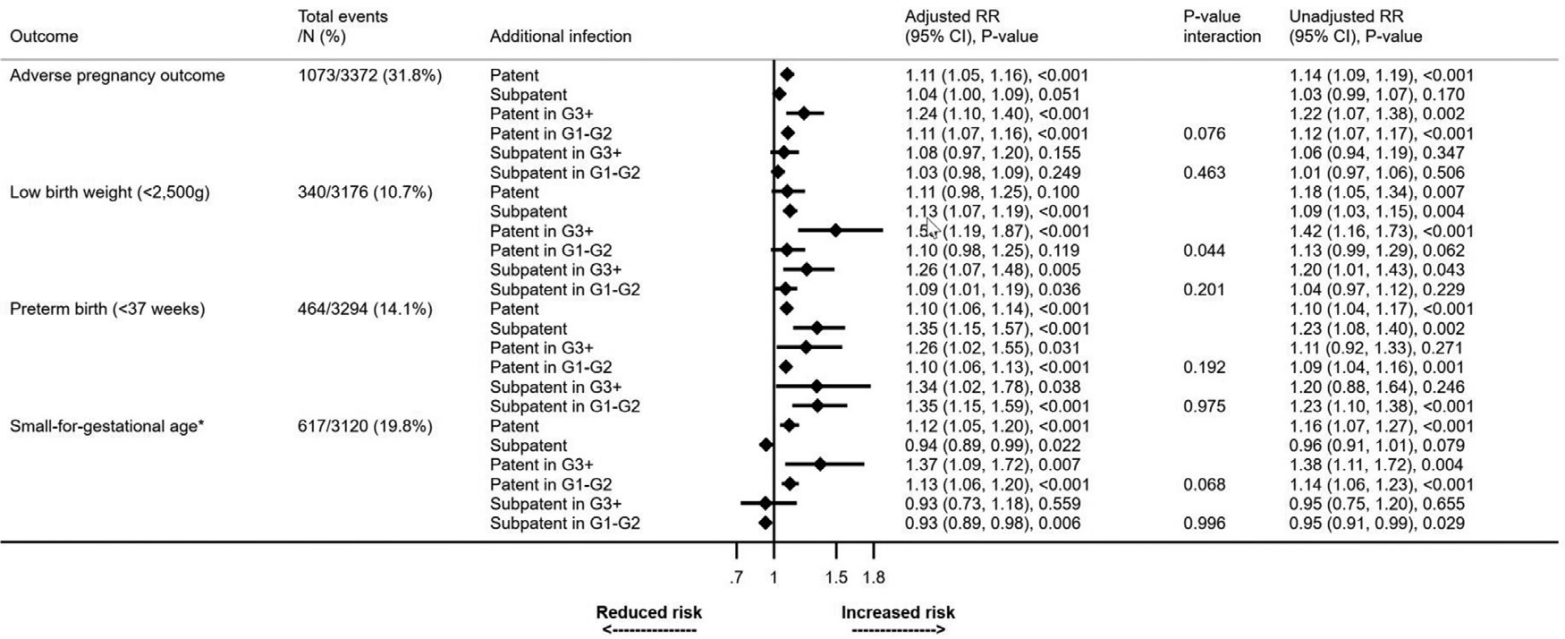
Impact of patent and subpatent infections on binary neonatal outcomes RR= risk ratio. For example, for the impact of patent infection on adverse pregnancy outcome, a value of 1.11 means an increase of 11% with each additional patent infection. The unadjusted risk ratio (RR) reflects models that include the stratification factor gravidity (primigravidae (G1) vs secundigravidae (G2) vs multigravidae (G3+)), which was used in some of the source studies, as well as site. The p-value for the interaction term reflects the difference in effect between gravidity strata. The adjusted models also include anaemia at enrolment (haemoglobin < 11 g/dL), gestational age (binary, study-specific median), and maternal ITN use at enrolment. * Small-for-gestational-age is defined as <10th percentile of the INTERGROWTH-21 reference population

**Figure 5:**
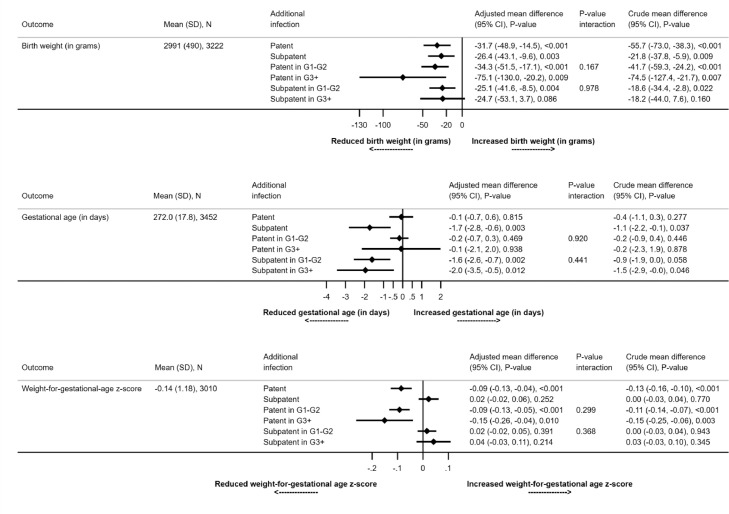
Impact of patent and subpatent infections on continuous neonatal outcomes *Mean change in gestational age at birth, weight-for-age z-score and corrected birth weight. For example, for the impact of patent infection on gestational age, a value of -0.07 means a reduction of 0.07 days in mean gestation age associated with each additional patent infection. The p-value for the interaction term reflects the difference in effect between gravidity strata. G1 = primigravidae; G2 = secundigravidae; G3+ = multigravidae.

Each patent infection during pregnancy was associated with an 11% increase in the risk of the composite adverse pregnancy outcome (adjusted [a]RR=1.11, 1.05-1.16, p<0.001). A smaller, non-significant increase was seen with subpatent infections (aRR=1.04, 1.00-1.09, p=0.05) (Figure 4). Each patent or subpatent infection was associated with a reduction in mean birthweight compared to women without infection (adjusted mean difference [a]MD: subpatent=32g, 15-49, p<0.001, patent=26g, 10-43, p=0.003) (Figure 5). Each subpatent infection was associated with a shortening of pregnancy duration by a mean of 1.7 days (95% CI 0.6-2.8, p=0.003). Patent infections were not associated with a shorter duration of pregnancy (aMD=0.1, -0.6-0.7, p=0.82). By contrast, patent (aMD=0.09, 0.04-0.13, p<0.001) but not subpatent infections (aMD=0.02, -0.06-0.02, p=0.25) were associated with lower mean weight-for-gestational-age Z-scores (Figure 5). Each subpatent infection was associated with an increased risk of LBW (aRR=1.13, 1.07-1.19, p<0.001) and preterm delivery (aRR=1.35, 1.15-1.57, p<0.001), but not SGA (Figure 4).

## 4 Discussion

This meta-analysis of five trials confirmed that ISTp with ACTs was not superior to the current IPTp-SP strategy in the situations where it was tested. These included areas in West Africa where the prevalence of SP resistance was low and in Kenya and Malawi where it was high. Babies born to mothers in the ISTp arm weighed slightly less than those in the IPTp-SP, though the risk of LBW was not significantly different. Women randomised to ISTp had a 21% increased risk of clinical malaria during pregnancy and an 18% increased risk of peripheral blood malaria infection at delivery, reflecting 31% more subpatent infections. There was no difference in the prevalence of placental malaria or anaemia between the two study arms. Further analysis to assess the impact of subpatent infections on newborn outcomes, while controlling for the effect of patent infections, showed that each antenatal subpatent infection was associated with a 26 gram decrease in mean birth weight and a 13% increased risk of LBW. This association with birthweight reflected an increased risk of preterm delivery rather than fetal growth retardation. Overall, these results suggest ISTp with the current generation of RDTs is not a suitable alternative to the existing IPTp-SP strategy, even in areas with high SP resistance in East and southern Africa. Our observation that subpatent infections are associated with an increased risk of LBW suggests that, compared with conventional RDT use for ISTp, the detection and treatment of more low-density infections with more sensitive diagnostic point-of-care tests may enhance birth outcomes. Further, the high proportion of women with evidence of malaria at delivery, including those in the IPTp-SP arm, highlights that better adherence to ITN usage and improved strategies to prevent malaria in pregnancy remain urgently needed to address the serious adverse effects of malaria in pregnancy on the developing fetus, newborn and infant development [2,32,33].

One reason for the modest efficacy of ISTp compared to IPTp-SP likely reflects a three- to six-fold difference (depending on the antimalarial used for ISTp) between the study arms in the duration of pregnancy spent under prophylaxis. Approximately one-quarter of women in the ISTp arms (26.4%) had at least one patent infection detected by RDTs during pregnancy. This proportion is a function of both malaria transmission intensity and the sensitivity of the diagnostic test, which is affected by parasite density and immunity. In the ISTp arm, only RDT-positive women received treatment and benefitted from the post-treatment prophylactic effect of the long-acting component of the ACT, estimated to be about two weeks with a 3-day treatment dose of artemether-lumefantrine and four weeks with dihydroartemisinin-piperaquine [34]. In contrast, women in the IPTp-SP arms received an average of 2.6 courses of SP. SP provides at least four weeks of prophylaxis against new infection in areas with sensitive parasites and about two weeks in high SP resistance areas such as in Malawi and western Kenya, where quintuple mutant parasites are saturated, but the sextuple mutants are still rare [3]. Additionally, because of the limited sensitivity of the current generation of RDTs, subpatent infections remain undetected and untreated and can persist for months, yet are cleared or suppressed with regular use of presumptive SP. These low-grade infections were associated with more LBW and preterm delivery independent of patent infections, i.e. they were observed even in women who never had a patent infection. The lack of effect on SGA suggests that the reductions in birthweight associated with subpatent infections are mediated primarily through preterm deliveries rather than fetal growth restriction. In contrast, patent infections were associated with both preterm delivery and SGA, suggesting that their effect on birth weight also results from fetal growth restriction. This relationship between patent infections and preterm delivery and SGA is not be restricted to highly malaria endemic areas and has also been described in areas with much lower malaria transmission along the Thailand-Myanmar border [35].

The failure of ISTp to show incremental effectiveness compared to IPTp-SP is consistent with a recent mathematical model based on these trials [30]. The model suggested that infections missed by standard RDTs lead to a greater proportion of inadequately treated infected women than does providing SP presumptively, i.e., the negative effects of misdiagnosed infections outweigh those of treatment and prophylaxis failures with SP in high SP resistance areas. However, the simulation also suggested that in high SP resistance settings, both IPTp-SP and ISTp, whilst failing to provide optimal protection, effectively prevent the majority of infections when compared to no intervention [30]. It remains to be determined if ISTp, or a hybrid of screen and treat approaches combined with IPTp-SP, may have a role in areas where SP is ineffective in clearing existing infections due to a high prevalence of parasites carrying the highly resistant ‘sextuple’ *dhfr/dhps* mutant haplotype that includes the *dhps*-A581G substitution [30]. As shown previously, the sensitivity of the current generation of RDTs was good at the first antenatal clinic visit, which occurred on average at 21 weeks gestation [30]. At that time, over 80% of infections, particularly in primigravidae, were detectable by RDTs. ISTp or single screening and treatment in pregnancy focusing on the first ANC, when the prevalence of malaria is highest and RDTs are most sensitive, may also be appropriate in areas of very low transmission where the exposure to malaria is low, but the adverse consequences of undetected infection remain high [36].

The observed difference in birthweight may also reflect a non-malaria effect of SP [37]. IPTp-SP reduces the risk of some sexually transmitted infections [38] and is associated with dose-dependent increases in birthweight [39] even in areas of low malaria transmission [40]. Three recent trials comparing IPTp with dihydroartemisinin-piperaquine (DP) versus SP, similarly noted that although women randomised to the IPTp-DP had substantially less malaria, infants born to women in the IPTp-SP arm had higher mean birthweights [12,13,41]. It was hypothesized that this reflected a potent non-malaria effect of IPTp-SP on birthweight [37]. The non-malarial mechanisms are still being elucidated, but potential mechanisms include SP's antimicrobial effect on pathogens or the gut or vaginal microbiome [37,42] or a potential anti-inflammatory effect, similar to that observed with cotrimoxazole [43].

A limitation of this study is that the effect of subpatent infection on birth outcome could only be adjusted for the number of patent infections, but not for their parasite density or duration. Furthermore, women were enrolled in the second or third trimester. Many women are already infected by their first antenatal visit, including from carriage of infections acquired prior to conception. Malaria infection early in pregnancy is associated with dysregulation of essential regulators of angiogenesis, metabolism, and inflammation resulting in placental insufficiency and an increased risk of maternal anaemia later in pregnancy, preterm birth, fetal growth restriction [44], [45], [46]. Preventing malaria earlier may reduce the risk that these infections persist, multiply, and sequester within the placenta at crucial stages of placental development. Modelling suggests that screening and treatment of RDT positive women with long-acting ACTs would have a substantial impact in early pregnancy when IPTp with SP is contraindicated [30]. Another limitation is the small number of studies included (N=5). For most outcomes, only 3 or 4 studies contributed. This precluded us from exploring the source of the heterogeneity in treatment effect, which was considerable for some of the outcomes.

As described in the primary study reports, ISTp was well tolerated. Although requiring more steps and more time, ISTp was also generally well received by providers and pregnant women [13,17,[47], [48], [49]]. Most women were amenable to regular testing for malaria and understood that asymptomatic infection could be harmful to the baby [48,49]. In East and southern Africa, providers generally feel that SP is no longer effective and therefore preferred to use an alternate antimalarial and considered it a benefit if this was limited to actively infected women. However, providers expressed doubts about the reliability of RDTs and had concerns that ISTp would not provide adequate protection [48]. In addition, both women and providers expressed concern that the 3-day duration of treatment might lead to poor adherence outside of a trial setting [48]. While none of these barriers are insurmountable, they should be considered when implementing screening strategies for malaria in pregnancy, e.g., as part of first-trimester screening or hybrid strategies with IPTp. The cost of ISTp is higher than IPTp. This should also be considered as the cost-effectiveness of ISTp improves when the efficacy of IPTp-SP drops [50]. The current five trials were all conducted in highly endemic settings, but as transmission drops, the number of women who may benefit from monthly IPTp falls. Under these lower transmission conditions, screen and treat strategies may be considered more acceptable than exposing large numbers of women to monthly antimalarial prophylaxis, especially if more complex 3-day IPTp regimens are used instead of single day SP [51].

This analysis of data from five trials found no evidence that ISTp with the current generation of RDTs improves maternal or neonatal outcomes compared to the current strategy IPTp with SP, and should not replace IPTp-SP even in areas with a high prevalence of parasites with the SP-resistant quintuple mutation. Further studies should be considered with highly sensitive diagnostic tests, as ISTp or as part of hybrid strategies that combine screening and treatment with IPTp-SP in high SP resistance areas. It remains possible that ISTp, ideally starting as early in pregnancy as possible, would perform more favourably in settings with a higher prevalence of the sextuple mutant resistant parasites or could be a potential strategy where the risk of malaria in pregnancy is low but its consequences still severe [52], [53], [54], [55], [56]. No IST studies have been conducted in such settings. In light of the high prevalence of malaria in the IPTp-SP arms, ^32^it is also critical to further explore IPT with more effective antimalarials such as dihydroartemisinin-piperaquine.

## Declaration of Competing Interest

All authors declare no competing interests.

## Data Availability

Individual patient data for studies contributing to this analysis are available from the Worldwide Antimalarial Resistance Network (WWARN) data repository.

## Appendix Supplementary materials

Image, application 1
