# Supplementary material for: Intermittent screening and treatment with artemisinin-combination therapy versus intermittent preventive treatment with sulphadoxine-pyrimethamine for malaria in pregnancy: a systematic review and individual participant data meta-analysis of randomised clinical trials
Source: eClinicalMedicine. 2021 Oct 25;41:101160. doi: 10.1016/j.eclinm.2021.101160 (PMC8556518; doi:10.1016/j.eclinm.2021.101160)
Supplement: Supplementary file 1 [file mmc1.pdf]

Supplementary appendix

Supplement to: Gutman et al., Intermittent screening and treatment with artemisinin-combination therapy versus intermittent preventive treatment with sulphadoxine-pyrimethamine for malaria in pregnancy: a systematic review and individual participant data meta-analysis of randomised clinical trials

Supplemental methods

Supplement 1: Eligibility criteria for trials

Trials were included if they were randomised controlled trials conducted in sub-Saharan Africa comparing intermittent preventive treatment with sulphadoxine-pyrimethamine (IPTp-SP) versus intermittent screening and treatment in pregnancy with an artemisinin-based therapy (ACT) (ISTp-ACT). Studies or study arms were excluded if they involved only HIV-infected women, used SP only for ISTp (because ACTs replaced SP as treatment drug in the general population and the 2nd and 3rd trimester of pregnancy), combined SP with other antimalarial drugs for IPTp, such as artemisinin derivatives or azithromycin, or with other interventions such IPTp with antimalarials other than SP.

Supplement 2: Quality and risk of bias assessment of trials

The risk of bias assessment for each included trial was conducted by two persons (JG and CK) using version 2 of the Cochrane risk-of-bias tool for randomised trials (RoB 2), which is structured into a fixed set of domains of bias, focusing on different aspects of trial design, conduct and reporting. A judgement about the risk of bias arising from each domain is proposed by an algorithm and can be overwritten by the authors with justification. Judgements can be 'Low', or 'High' risk of bias, or can express 'Some concerns'.[1] Where disagreement occurred, a joint review of the study was conducted until agreement was reached by consensus. Studies were not excluded a-priori based on their quality score.

Supplement 3: Definition of outcomes

Primary outcomes

- Malaria at delivery: Any *Plasmodium* infection detected in peripheral or placental blood by PCR, microscopy, RDT or histopathology (acute and/or chronic infections)
- Adverse pregnancy outcome: A composite of low birth weight (<2500 grams), small-for-gestational-age (SGA, <10th percentile relative to INTERGROWTH-21st gender-specific chart),[2] or and preterm delivery (<37 weeks gestation).

Secondary maternal outcomes

- Clinical malaria: The definition was based on the definition used in the source studies, as this defined when treatment was given. In Tagbor 2010, clinical malaria was defined as any positive RDT documented in a woman who presented with a history of fever or other features suggestive of malaria.[3] In Desai and Madanitsa,[4,5] clinical malaria was defined by a positive RDT or smear in conjunction with documented fever (>37.5 ^{o}C) or reported fever in the past 24 hours. In Tagbor 2015, any positive RDT plus documented fever (>37.5 ^{o}C) was considered clinical malaria.[6] Esu did not report on clinical malaria and was not included in the analysis of this outcome.[7]
- Subpatent malaria during pregnancy: *Plasmodium* infection during pregnancy identified in the maternal peripheral blood by positive PCR, with negative microscopy and/or RDT
- Subpatent malaria at delivery: *Plasmodium* infection at delivery identified in the maternal peripheral blood by positive PCR with negative microscopy and/or RDT
- Patent malaria at delivery: *Plasmodium* infection during pregnancy identified in the maternal peripheral blood by positive microscopy or RDT, with PCR either positive or missing

The analyses of the impact of ISTp versus IPTp excluded enrolment and delivery time points from the incidence data during pregnancy. For the analysis of the impact of patent and subpatent infection on adverse pregnancy outcomes, the enrolment and delivery time points were included.

Secondary newborn outcomes

- Low birth weight (LBW): A birthweight < 2,500g measured. All birthweights in the analyses refer to corrected birthweights taken within seven days (168 hours) after birth. Birthweights taken more than 24 hours after delivery were corrected for the physiological fall in birth weight in breastfed infants occurring in the first days following delivery.[8,9] Birth weights taken 24-48h hours, 48-72 hours, 72-96 hours, 96-120 hours, 120-144 hours and 144-168 hours after delivery were corrected by a factor +5%, +5.4%, +3.4%, +1.3%, +0% and -1.2%, respectively to obtain the estimated weight at birth.[10,11]
- Preterm birth (PTD): A gestational age at birth of < 37 weeks. Gestational age at birth was defined as follows: In the trial in Malawi, gestational age at birth was based on the gestational age at enrolment assessed by ultrasound.[5] In the three other IPD studies in Kenya[4] and West-Africa[3,6] gestational at birth was based on physical parameters assessed at birth using the Ballard score.[12]
- Small-for-gestational-age: A birthweight <10th percentile of the INTERGROWTH-21st reference standard which is derived from a multi-ethnic cohort of low-risk, well-nourished mothers with uncomplicated pregnancies.[2,13] Infants with a gestational age >42 weeks were classified as SGA if they were lighter than the 42-week reference standard; those who were heavier than the cut-off for SGA at 42 weeks were excluded from the analysis.
- Spontaneous miscarriage: Loss of foetus before 28 weeks gestational age
- Stillbirth: Loss of foetus at or after 28 weeks gestation or birth of foetus showing no signs of life
- Foetal loss: Stillbirth or miscarriage
- Perinatal death: stillbirths and deaths in the first week of life
- Neonatal death: death within 28 days of birth
- Infant mortality: Death of a live-born infant by 6-8 weeks of age
- Congenital anomalies: Physical abnormality of infant detected at delivery or newly noted abnormality during the infant visits.

Risk ratios were measured for the binary outcomes, and mean differences for continuous outcomes. Missing outcomes were not imputed, nor missing covariates.

Supplement 4: Model selected and assessment of heterogeneity

Fixed-effect models were used because only five trials contributed, and heterogeneity cannot be reliably estimated with a small number of studies (3 to 4 for most outcomes), resulting in poor precision of the estimate of the between-studies variance.[14-16] The extent of heterogeneity was measured using the I^2 statistic,[17] which is a measure of the proportion of total variability explained by heterogeneity rather than chance expressed as a percentage, with 0-40% representing no or little heterogeneity, 30-60% moderate heterogeneity, 50-90% substantial heterogeneity, and 75-100% considerable heterogeneity.[18]

Supplement 5: Analytic methods for assessing the effect of exposure to subpatent malaria on pregnancy outcomes

The effect of exposure to subpatent malaria on pregnancy outcomes was examined using fixed-effect models with Poisson regression for binary outcomes and linear regression for continuous outcomes, accounting for study and number of subpatent and patent tests conducted. Data from patients in both ISTp and IPTp-SP were included in the analysis as long as there was data from at least one test for subpatent infection and one for patent infection. Robust Huber/White/sandwich estimate of variance was used. Poisson regression is a generalized linear model with a log link and a

Poisson distribution. When the outcome is binary, the exponentiated coefficients are risk ratios instead of incidence-rate ratios.[19] The two exposure variables of interest, the number of patent infections and the number of subpatent infections, were modelled as continuous variables. In the binary models, risk ratios for these variables correspond to the change in the risk of the adverse outcome associated with one additional positive test (malaria infection) during pregnancy. In models with continuous outcomes, the mean difference in the outcome measure associated with each additional positive test (malaria infection) was estimated. Fractional polynomials were also used to explore nonlinear relationships between number of patent and subpatent infections and pregnancy outcomes and were considered if the reduction in deviance compared to the linear model was significant at p<0.05. The results of linear models are presented throughout because the differences with non-linear models were non-significant for both variables and all outcomes.

Crude models included the two exposures of interest and study arm. Adjusted models also included gestational age at enrolment, maternal age, gravidity (G1-G2/G3+) and the number of sick visits. Adjusted models were used as the primary analysis to assess the impact of patent and subpatent infections because the exposure groups of interested were not randomised.

A previous analysis of the study in Malawi[20] looked at the effect of subpatent infections among the women enrolled in the ISTp arm and found that among women of all gravidities, cumulative subpatent infections were not associated with an increased risk of LBW (RR= 0.78, 95% CI: 0.38–1.60) nor preterm delivery (RR=1.35, 95% CI: 0.87-2.1) compared to women with no infection. By contrast, the current analysis found that subpatent infections were associated with an increased risk of LBW and preterm delivery (aRR= 1.13, 95% CI: 1.07-1.19 and aRR= 1.35, 95% CI: 1.15-1.57, respectively). The previous analysis had less power to detect the effect of subpatent infections. In addition to the smaller sample size, it included only RDT positives and defined subpatent infection as 'any woman with at least one RDT-negative and PCR-positive infection' vs no infection. The current analysis used both RDT and microscopy to define patent and sub patent infections. Furthermore, in the previous analysis, only women in the ISTp arm were included, whereas, in the current analysis, both arms were included in the analysis of the impact of patent and subpatent infections.

Supplement 6: Sensitivity analysis of the impact of the method of assessment for gestational age

We conducted a sensitivity analysis to explore whether the method of assessment influenced the effect of subpatent parasitaemia because the definition of preterm and SGA depends on the accuracy of gestational age assessment and because of the substantially higher accuracy of gestational age dating by ultrasound compared to other methods (i.e., fundal height, Ballard scores[12], last menstrual period).[21] This could be done using the data from the study in Malawi, which had used both early ultrasound measurement at enrolment and the Ballard score at delivery.[5] For this study, we used the INTERGROWTH-21st reference group[22] to assess SGA, while the original publications used Schmiegelow[23] (for Desai, 2015 and Madanitsa, 2016) and Landis[24] (for Tagbor, 2015).

Supplemental results

Supplement 7: List of excluded studies

1. Community-based Malaria Screening and Treatment for Pregnant Women Receiving Standard Intermittent Preventive Treatment With Sulfadoxine-Pyrimethamine: A Multicenter (The Gambia, Burkina Faso, and Benin) Cluster-randomized Controlled Trial. Clin Infect Dis 2019; 68(4): 586-96.

2. Agan T, Ekabua J, Udoh A, Ekanem E, Efiok E, Mgbekem M. Prevalence of anemia in women with asymptomatic malaria parasitemia at first antenatal care visit at the University of Calabar Teaching Hospital, Calabar, Nigeria. International Journal of Women's Health 2010; 2: 229-33.
3. Agboli E. Malaria and anaemia in pregnant and non-pregnant women of child-bearing age at the University Hospital-KNUST, Kumasi; 2011.
4. Ahmed, R, Poespoprodjo, et al. Efficacy and safety of intermittent preventive treatment and intermittent screening and treatment versus single screening and treatment with dihydroartemisinin-piperaquine for the control of malaria in pregnancy in Indonesia: a cluster-randomised, open-la. Lancet Infectious Diseases 2019; 19(9): 973-87.
5. Ahmed R, Asih PPB, Noviyanti R, et al. The clinical burden of microscopically patent and sub-microscopic P. falciparum and P. vivax malaria in pregnancy in Indonesia. 2014; 2014. p. P2.
6. Ahmed R, Poespoprodjo JR, Syafruddin D, et al. Efficacy and safety of intermittent preventive treatment and intermittent screening and treatment versus single screening and treatment with dihydroartemisinin-piperaquine for the control of malaria in pregnancy in Indonesia: a cluster-randomised, open-label, superiority trial. Lancet Infect Dis 2019; 19(9): 973-87.
7. Ahorlu CK, Koram KA, Seakey AK, Weiss MG. Effectiveness of combined intermittent preventive treatment for children and timely home treatment for malaria control. Malar J 2009; 8: 292.
8. Almond D, Madanitsa M, Mwapasa V, et al. Provider and user acceptability of intermittent screening and treatment for the control of malaria in pregnancy in Malawi. Malar J 2016; 15(1): 574.
9. Amimo, F, Moon, et al. Trends in comparative efficacy and safety of malaria control interventions for maternal and child health outcomes in Africa: a study protocol for a Bayesian network meta-regression exploring the effect of HIV and malaria endemicity spectrum. BMJ open 2019; 9(2): e024313.
10. Anthony, A. Characterizing functional antibody responses in malaria in pregnancy; 2019.
11. Asweto CO. Disparities in quality of antenatal clinic care in Kenya: Analysis of Kenya Demographic Health Survey 2008/9. 2015; 2015.
12. Awine T, Belko MM, Oduro AR, et al. The risk of malaria in Ghanaian infants born to women managed in pregnancy with intermittent screening and treatment for malaria or intermittent preventive treatment with sulfadoxine/pyrimethamine. Malaria Journal 2016; 15(1): 46.
13. Basra A, Mombo-Ngoma G, Melser MC, et al. Efficacy of mefloquine intermittent preventive treatment in pregnancy against Schistosoma haematobium infection in Gabon: a nested randomized controlled assessor-blinded clinical trial. Clin Infect Dis 2013; 56(6): e68-75.
14. Brieger W. Evolution of intermittent screening and treatment for malaria in pregnancy control. Africa Health 2012; July 2012: 33-5.
15. Cairns M, Woukeu A, Tagbor H. Screening and treatment of malaria in pregnancy in West Africa, 2015.
16. Chadewa J, Bishanga D, Roman E, et al. Using the antenatal care quality improvement tool and targeted training to strengthen ANC services including MIP in Kagera and Mara regions, Tanzania. 2017; 2017.
17. Chaponda EB. The epidemiology of malaria, curable sexually transmitted and reproductive tract infections and their coinfection among pregnant women in a catchment area in Nchelenge District, Zambia; 2017.

18. Chico RM, Chandramohan D. Intermittent preventive treatment of malaria in pregnancy: at the crossroads of public health policy. Tropical Medicine and International Health 2011; 16(7): 774-85.
19. Chico RM, Moss WJ. Prevention of malaria in pregnancy: a fork in the road? Lancet 2015; 386(10012): 2454-6.
20. Clerk CA, Bruce J, Affipunguh PK, et al. A randomized, controlled trial of intermittent preventive treatment with sulfadoxine-pyrimethamine, amodiaquine, or the combination in pregnant women in Ghana. J Infect Dis 2008; 198(8): 1202-11.
21. Correa G, Das M, Kovelamudi R, et al. High burden of malaria and anemia among tribal pregnant women in a chronic conflict corridor in India. Conflict and Health 2017; 11: 10.
22. Cosmic C. Community-based malaria Screening and treatment for pregnant women receiving standard intermittent preventive treatment with sulfadoxine-pyrimethamine: A multicentre (The Gambia, Burkina Faso and Benin) cluster randomised controlled trial. Clinical Infectious Diseases 2018; 68(4): 586-96.
23. Dassah ET, Adu-Sarkodie Y, Mayaud P. Estimating the uptake of maternal syphilis screening and other antenatal interventions before and after national rollout of syphilis point-of-care testing in Ghana. International Journal of Gynaecology and Obstetrics 2015; 130 Suppl 1: S63-S9.
24. Dassah ET, Adu-Sarkodie Y, Mayaud P. Factors associated with failure to screen for syphilis during antenatal care in Ghana: a case control study. BMC Infectious Diseases 2015; 15(1): 125.
25. Desai M, Gutman J, L'Lanziva A, et al. Intermittent screening and treatment or intermittent preventive treatment with dihydroartemisinin-piperaquine versus intermittent preventive treatment with sulfadoxine-pyrimethamine for the control of malaria during pregnancy in western Kenya: an open-label, three-group, randomised controlled superiority trial. Lancet 2015; 386(10012): 2507-19.
26. Desai M, Hill J, Fernandes S, et al. Prevention of malaria in pregnancy. Lancet Infectious Diseases 2018; 18(4): e119-e32.
27. Esu EB. The effectiveness of intermittent screening and treatment with artemether-lumefantrine for malaria prevention in pregnancy in South East Nigeria; 2017.
28. Fernandes S, Sicuri E, Halimatou D, et al. Cost effectiveness of intermittent screening followed by treatment versus intermittent preventive treatment during pregnancy in West Africa: analysis and modelling of results from a non-inferiority trial. Malaria Journal 2016; 15(1): 493.
29. Fryauff DJ, McCoy AJ, Atugaba F, et al. Does enhanced detection and analysis of malaria infections in umbilical cord blood samples explain low birth weight and fetal anaemia in newborns of the Kassena-Nankana District of North Eastern Ghana? ; 2010; 2010. p. 282-.
30. Garg, S, Dewangan, M, Barman, O. Malaria prevalence in symptomatic and asymptomatic pregnant women in a high malaria-burden state in India. Tropical Medicine and Health 2020; 48: 71.
31. Global Malaria P, World Health O. Intermittent screening and treatment in pregnancy and the safety of ACTs in the first trimester, 2015.
32. Gnaneswaran, B, Conroy, et al. Does malaria in pregnancy affect neurodevelopmental outcomes? ; 2019; 2019. p. S108.
33. Goldenberg RL, McClure EM, Saleem S, Reddy UM. Infection-related stillbirths. Lancet 2010; 375(9724): 1482-90.

34. González R, Desai M, Macete E, et al. Intermittent preventive treatment of malaria in pregnancy with mefloquine in HIV-infected women receiving cotrimoxazole prophylaxis: a multicenter randomized placebo-controlled trial. PLoS Med 2014; 11(9): e1001735.
35. González R, Mombo-Ngoma G, Ouédraogo S, et al. Intermittent preventive treatment of malaria in pregnancy with mefloquine in HIV-negative women: a multicentre randomized controlled trial. PLoS Med 2014; 11(9): e1001733.
36. Group WHOER. Malaria in pregnancy, 2016.
37. Gutman J, L'Lanziva J, Otieno K, et al. Subpatent malaria infection is not associated with poor birth outcomes in Kenyan women receiving intermittent screening and treatment or intermittent preventive treatment for malaria in pregnancy. 2016; 2016. p. 7-.
38. Hellewell, J, Walker, et al. Using ante-natal clinic prevalence data to monitor temporal changes in malaria incidence in a humanitarian setting in the Democratic Republic of Congo. Malaria Journal 2018; 17: 312.
39. Hill, J, Ouma, et al. Intermittent screening and treatment with dihydroartemisinin-piperaquine for the prevention of malaria in pregnancy: implementation feasibility in a routine healthcare system setting in western Kenya. Malaria Journal 2020; 19(1): 433.
40. Hill J, Hoyt J, Achieng F, et al. User and Provider Acceptability of Intermittent Screening and Treatment and Intermittent Preventive Treatment with Dihydroartemisinin-Piperaquine to Prevent Malaria in Pregnancy in Western Kenya. PLoS One 2016; 11(3): e0150259.
41. Hopkins H, Cunningham J, Zongo I, et al. HRP2 and PLDH RDTs compared with microscopy, PCR and histology for detection of placental malaria during pregnancy and at delivery in areas of varied transmission. 2012; 2012. p. 395-.
42. Hounkonnou, C P, Tuikue N, et al. Sub-optimal intermittent preventive treatment in pregnancy administration increases the risk of submicroscopic Plasmodium falciparum infection in pregnant women: A preconception cohort study in Benin. 2020; 2020. p. 216.
43. Hounkonnou, C. P A, Ndam, et al. Sub-optimal intermittent preventive treatment in pregnancy (IPTp) is associated with an increased risk of submicroscopic P. falciparum infection in pregnant women: a prospective cohort study in Benin. Clinical Infectious Diseases 2020.
44. Hoyt J, Landuwulang CUR, Ansariadi, et al. Intermittent screening and treatment or intermittent preventive treatment compared to current policy of single screening and treatment for the prevention of malaria in pregnancy in Eastern Indonesia: acceptability among health providers and pregnant women. Malar J 2018; 17(1): 341.
45. Huijben S, Macete E, Mombo-Ngoma G, et al. Counter-Selection of Antimalarial Resistance Polymorphisms by Intermittent Preventive Treatment in Pregnancy. J Infect Dis 2020; 221(2): 293-303.
46. Hyacinth HL, Oguche S, Yilgwan CS. Summary description of 24 cases of neonatal malaria seen at a tertiary health center in Nigeria. Iranian Journal of Pediatrics 2012; 22(1): 87-91.
47. Ishaque S, Yakoob MY, Imdad A, Goldenberg RL, Eisele TP, Bhutta ZA. Effectiveness of interventions to screen and manage infections during pregnancy on reducing stillbirths: a review. BMC Public Health 2011; 11 Suppl 3: S3.
48. Jafari G, S, Accrombessi, et al. Malaria in pregnancy: from pre-conception to early pregnancy, a genotyping analysis. 2018; 2018. p. 325.
49. Kesteman T, Randrianarivelojosia M, Piola P, Rogier C. Post-deployment effectiveness of malaria control interventions on Plasmodium infections in Madagascar: a comprehensive phase IV assessment. Malaria Journal 2016; 15: 322.

50. Khamis MIR, Roman E, Stevenson R, et al. IPTp policy in Zanzibar towards pre-elimination of malaria: results from a study of placental parasitemia. 2013; 2013.
51. Kimani J, Phiri K, Kamiza S, et al. Azithromycin/chloroquine (AZCQ) versus sulfadoxine/pyrimethamine (SP) in intermittent preventive treatment of falciparum malaria infection in pregnant women (IPTp) in Sub-Saharan Africa: an open-label randomized trial. 2015; 2015.
52. Kitojo, C, Chacky, et al. Evaluation of a single screen and treat strategy to detect asymptomatic malaria among pregnant women from selected health facilities in Lindi region, Tanzania. Malaria Journal 2020; 19(1): 438.
53. Kuepfer, I, Mishra, et al. Effectiveness of intermittent screening and treatment for the control of malaria in pregnancy: a cluster randomised trial in India. BMJ Global Health 2019; 4(4): e001399.
54. Kyabayinze DJ, Zongo I, Cunningham J, et al. HRP2 and pLDH-based rapid diagnostic tests, expert microscopy, and PCR for detection of malaria infection during pregnancy and at delivery in areas of varied transmission: A prospective cohort study in Burkina Faso and Uganda. PLoS One 2016; 11(7): e0156954.
55. Landis SH, Lokomba V, Ananth CV, et al. Impact of maternal malaria and under-nutrition on intrauterine growth restriction: a prospective ultrasound study in Democratic Republic of Congo. Epidemiology and Infection 2009; 137(2): 294-304.
56. Lee PW, Liu CT, do Rosario VE, de SB, Rampao HS, Shaio MF. Potential threat of malaria epidemics in a low transmission area, as exemplified by Sao Tome and Principe. Malaria Journal 2010; 9: 264.
57. Levitt B, Madanitsa M, Ter Kuile FO, et al. Comparative impacts of antenatal malaria prevention strategies on Plasmodium falciparum SP-resistance alleles in Malawi. American Journal of Tropical Medicine and Hygiene 2016; 95(S5). p. 400.
58. Lin JT, Mbewe B, Taylor SM, Luntamo M, Meshnick SR, Ashorn P. Increased prevalence of dhfr and dhps mutants at delivery in Malawian pregnant women receiving intermittent preventive treatment for malaria. Tropical Medicine and International Health 2013; 18(2): 175-8.
59. Madani FAE. Study of malaria among pregnant women admitted to Sinnar Teaching Hospital: Proportion and risk factors; 2011.
60. Madanitsa, MM. The potential of intermittent screening and treatment with dihydroartemisinin-piperaquine for the control of malaria in pregnancy in areas with high sulphadoxine-pyrimethamine resistance; 2015.
61. Madanitsa M, Kalilani L, Mwapasa V, et al. Scheduled intermittent screening with rapid diagnostic tests and treatment with dihydroartemisinin-piperaquine versus intermittent preventive therapy with sulfadoxine-pyrimethamine for malaria in pregnancy in Malawi: An open-label randomized controlled tr. PLoS Medicine 2016; 13(9): e1002124.
62. Madanitsa M, Kalilani-Phiri L, Mwapasa V, et al. Scheduled screening versus preventive treatment for the control of malaria in pregnancy in Malawi: a randomized controlled trial. 2014; 2014. p. 206-.
63. Manirakiza A. Use of rapid diagnostic test (paracheck-pf©) to improve malaria treatment in antenatal clinics in Bangui, Central African Republic; 2012.
64. Manirakiza A, Sepou A, Serdouma E, et al. Effectiveness of two antifolate prophylactic strategies against malaria in HIV-positive pregnant women in Bangui, Central African Republic: study protocol for a randomized controlled trial (MACOMBA). Trials 2013; 14: 255.

65. Mbonye AK, Magnussen P. Symptom-based diagnosis of malaria and its implication on antimalarial drug use in pregnancy in Central Uganda: results from a community trial. International Journal of Adolescent Medicine and Health 2010; 22(2): 257-62.
66. Menendez C, D'Alessandro U, ter Kuile FO. Reducing the burden of malaria in pregnancy by preventive strategies. Lancet Infectious Diseases 2007; 7(2): 126-35.
67. Menezes EV, Yakoob MY, Soomro T, Haws RA, Darmstadt GL, Bhutta ZA. Reducing stillbirths: prevention and management of medical disorders and infections during pregnancy. BMC Pregnancy and Childbirth 2009; 9 Suppl 1: S4.
68. Mens P, Schallig H. The COSMIC consortium community-based scheduled screening and treatment of malaria in pregnancy for improved maternal and infant health. 2013; 2013. p. 146-.
69. The Nambia Ministry of Health and Social Services (MoHSS) and ICF International. 2014. The Namibia Demographic and Health Survey 2013. Windhoek, Namibia, and Rockville, Maryland, USA: MoHSS and ICF International.
70. Minja DT, Schmiegelow C, Mmbando B, et al. Plasmodium falciparum mutant haplotype infection during pregnancy associated with reduced birthweight, Tanzania. Emerging Infectious Diseases 2013; 19(9).
71. Minja DT, Schmiegelow C, Oesterholt M, et al. Reliability of rapid diagnostic tests in diagnosing pregnancy-associated malaria in north-eastern Tanzania. Malaria Journal 2012; 11(1): 211.
72. Moneke-Anyanwoke N, Mwesigwa J, Affara M, et al. Effect of community-based scheduled screening and treatment (CSST) of malaria in pregnancy on infant malaria infection in a seasonal malaria transmission setting. 2017; 2017. p. A29-A30.
73. Moorthy, D, Merrill, et al. The impact of nutrition-specific and nutrition-sensitive interventions on hemoglobin concentrations and anemia: A meta-review of systematic reviews. Advances in Nutrition 2020; 11(6): 1631-45.
74. Moya-Alvarez V, Abellana R, Cot M. Pregnancy-associated malaria and malaria in infants: an old problem with present consequences. Malaria Journal 2014; 13(1): 271.
75. Msyamboza K, Senga E, Tetteh-Ashong E, Kazembe P, Brabin BJ. Estimation of effectiveness of interventions for malaria control in pregnancy using the screening method. International Journal of Epidemiology 2007; 36(2): 406-11.
76. Mubyazi GM, Magnussen P, Goodman C, et al. Implementing intermittent preventive treatment for malaria in pregnancy: review of prospects, achievements, challenges and agenda for research. Open Tropical Medicine Journal 2008; 1: 92-100.
77. Muhindo MK, Jagannathan P, Kakuru A, et al. Intermittent preventive treatment with dihydroartemisinin-piperaquine and risk of malaria following cessation in young Ugandan children: a double-blind, randomised, controlled trial. Lancet Infect Dis 2019; 19(9): 962-72.
78. Nakelembe M, Kyabayinze D, Compaore Y, et al. Detection of placental malaria and impact of RDT screening and treatment on pregnancy outcomes in areas of varied transmission. 2012; 2012. p. 138-.
79. Natama HM, Rovira-Vallbona E, Sorgho H, et al. Additional screening and treatment of malaria during pregnancy provides further protection against malaria and nonmalarial fevers during the first year of life. Journal of Infectious Diseases 2018; 217(12): 1967-76.
80. Natama MH, Rovira-Valbona E, Sorgho H, et al. Community-based scheduled screening and treatment of malaria during pregnancy provides additional protection against febrile illnesses during the first year of life in a birth cohort study in Burkina Faso. 2017; 2017.

81. Nnaji GA, Ikechebelu JI. An evaluation of the use of reported febrile illness in predicting malaria in pregnancy. Journal of Obstetrics and Gynaecology 2007; 27(8): 791-4.
82. Nwaneri DU, Adeleye OA, Ande AB. Asymptomatic malaria parasitaemia using rapid diagnostic test in unbooked pregnant women in rural Ondo-south district, Nigeria. Journal of Preventive Medicine and Hygiene 2013; 54(1): 49-52.
83. Nwogu I, L.E. Malaria in pregnancy (MiP): assessing Communities' response to community SST (CSST) carried out by CHW; 2018.
84. Olaleye, A, Okusanya, et al. A systematic review and meta-analysis of dihydroartemisinin-piperaquine versus sulphadoxine-pyrimethamine for malaria prevention in pregnancy. International journal of Gynaecology and Obstetrics 2019; 146(1): 43-55.
85. Ononge S, Campbell O, Mirembe F. Haemoglobin status and predictors of anaemia among pregnant women in Mpigi, Uganda. BMC Res Notes 2014; 7: 712.
86. Orish VN, Onyeabor OS, Boampong JN, et al. Prevalence of intermittent preventive treatment with sulphadoxine-pyrimethamine (IPTp-SP) use during pregnancy and other associated factors in Sekondi-Takoradi, Ghana. African Health Sciences 2015; 15(4): 1087-96.
87. Paintain L, Hill J, Ahmed R, et al. Cost-effectiveness of intermittent preventive treatment with dihydroartemisinin-piperaquine versus single screening and treatment for the control of malaria in pregnancy in Papua, Indonesia: a provider perspective analysis from a cluster-randomised trial. Lancet Glob Health 2020; 8(12): e1524-e33.
88. Pell C, Meñaca A, Chatio S, Hodgson A, Tagbor H, Pool R. The acceptability of intermittent screening and treatment versus intermittent preventive treatment during pregnancy: results from a qualitative study in Northern Ghana. Malar J 2014; 13: 432.
89. Phiri K, Kimani J, Mtove GA, et al. Parasitological Clearance Rates and Drug Concentrations of a Fixed Dose Combination of Azithromycin-Chloroquine in Asymptomatic Pregnant Women with Plasmodium Falciparum Parasitemia: An Open-Label, Non-Comparative Study in Sub-Saharan Africa. PLoS One 2016; 11(11): e0165692.
90. Plotkin M, Roman E, Lacoste M. Low levels of placental parasitemia among women delivering in health facilities in Zanzibar: policy implications for IPTp. 2013; 2013.
91. Plotkin M, Said K, Hendler N, et al. Low prevalence of placental malaria infection among pregnant women in Zanzibar: policy implications for IPTp. 2012; 2012. p. 266-.
92. President's Malaria Initiative. Rwanda Malaria Operational Plan FY 2013, 2013.
93. President's Malaria Initiative. Rwanda Malaria Operational Plan FY2018, 2018.
94. President's Malaria Initiative. Rwanda Malaria Operational Plan FY2019, 2019.
95. President's Malaria Initiative. Tanzania Malaria Operational Plan FY 2014, 2014.
96. President's Malaria Initiative. Tanzania Malaria Operational Plan FY 2015, 2015.
97. President's Malaria Initiative. Tanzania Malaria Operational Plan FY 2016, 2016.
98. President's Malaria Initiative. Tanzania Malaria Operational Plan FY2017, 2017.
99. President's Malaria Initiative. Tanzania Malaria Operational Plan FY2018, 2018.
100. President's Malaria Initiative. Tanzania Malaria Operational Plan FY2019, 2019.
101. President's Malaria Initiative. President's Malaria Initiative Technical Guidance, 2016.
102. Rogerson SJ, Unger HW. Prevention and control of malaria in pregnancy - new threats, new opportunities? Expert Review of Anti-infective Therapy 2016.
103. Ross AB, DeStigter KK, Coutinho A, et al. Ancillary benefits of antenatal ultrasound: an association between the introduction of a low-cost ultrasound program and an increase in the numbers of women receiving recommended antenatal treatments. BMC Pregnancy and Childbirth 2014; 14(1): 424.
104. Ruizendaal E. Malaria in pregnancy: in search of tools for improved prevention (2017); 2017.

105. Ruizendaal E, Schallig H, Scott S, et al. Evaluation of Malaria Screening during Pregnancy with Rapid Diagnostic Tests Performed by Community Health Workers in Burkina Faso. Am J Trop Med Hyg 2017; 97(4): 1190-7.
106. Schallig H, Ruizendaal E, Traore M, et al. Screening for malaria in pregnancy with RDTs by community health workers in Nanoro, Burkina Faso. 2017; 2017.
107. Scott S, Mens PF, Tinto H, et al. Community-based scheduled screening and treatment of malaria in pregnancy for improved maternal and infant health in The Gambia, Burkina Faso and Benin: study protocol for a randomized controlled trial. Trials 2014; 15: 340.
108. Shah, M.P, Choudhary, et al. The effectiveness of intermittent preventive treatment in pregnancy with alternative antimalarials compared to sulfadoxine-pyrimethamine for the prevention of low birth weight: A systematic review and meta-analysis. 2018; 2018. p. 120.
109. Singh N, Singh MP, Wylie BJ, et al. Malaria prevalence among pregnant women in two districts with differing endemicity in Chhattisgarh, India. Malaria Journal 2012; 11(1): 274.
110. Smith LA, Jones C, Adjei RO, et al. Intermittent screening and treatment versus intermittent preventive treatment of malaria in pregnancy: user acceptability. Malar J 2010; 9: 18.
111. Smith PL, Antwi GD, Jones C, et al. Intermittent screening and treatment versus intermittent preventive treatment of malaria in pregnancy: provider knowledge and acceptability. PLoS ONE 2011; 6(8): e24035.
112. Stephens JK, Ofori MF, Quakyi IA, Wilson ML, Akanmori BD. Prevalence of peripheral blood parasitaemia, anaemia and low birthweight among pregnant women in a suburban area in coastal Ghana. Pan African Medical Journal 2014; 17 Suppl 1: 3.
113. Tadesse, G, Kamaliddin, et al. Active case detection of malaria in pregnancy using loop-mediated amplification (LAMP): a pilot outcomes study in South West Ethiopia. Malaria Journal 2020; 19(1): 305.
114. Taylor SM, Levitt B, Freedman B, et al. Interactions Between Antenatal Sulfadoxine-Pyrimethamine, Drug-Resistant Plasmodium falciparum Parasites, and Delivery Outcomes in Malawi. J Infect Dis 2020; 222(4): 661-9.
115. Tegegne B, Getie S, Lemma W, Mohon AN, Pillai DR. Performance of loop-mediated isothermal amplification (LAMP) for the diagnosis of malaria among malaria suspected pregnant women in Northwest Ethiopia. Malaria Journal 2017; 16(1): 34.
116. Teo A. Malarial antibodies to Plasmodium falciparum during pregnancy in times of decreasing malaria prevalence; 2015.
117. Teo A, Randall LM, Madanitsa M, et al. Intermittent screening and treatment with dihydroartemisinin-piperaquine and intermittent preventive therapy with sulfadoxine-pyrimethamine have similar effects on malaria antibody in pregnant Malawian women. Sci Rep 2019; 9(1): 7878.
118. Tetteh-Ashong E. Evaluation of a screening method to assess the efficacy of intermittent preventive treatment with SP in pregnant women in Malawi; 2005.
119. Umbers AJ, Unger HW, Rosanas-Urgell A, et al. Accuracy of an HRP-2/panLDH rapid diagnostic test to detect peripheral and placental Plasmodium falciparum infection in Papua New Guinean women with anaemia or suspected malaria. Malaria Journal 2015; 14(1): 412.
120. Unger HW, Rosanas-Urgell A, Robinson LJ, et al. Microscopic and submicroscopic Plasmodium falciparum infection, maternal anaemia and adverse pregnancy outcomes in Papua New Guinea: a cohort study. Malar J 2019; 18(1): 302.
121. Vasquez, A M, Zuluaga, et al. Diagnostic accuracy of loop-mediated isothermal amplification (LAMP) for screening malaria in peripheral and placental blood samples from pregnant women in Colombia. Malaria journal 2018; 17(1): 262.

122. Volker F, Cooper P, Bader O, et al. Prevalence of pregnancy-relevant infections in a rural setting of Ghana. BMC Pregnancy and Childbirth 2017; 17(1): 172.
123. Walker, P, Cairns, et al. Using routine antenatal-care based RDT testing to measure population transmission: the key role of pregnancy-specific patterns of susceptibility and immunity. 2018; 2018. p. 327-8.
124. Walker, P. G T, Cairns, et al. Modelling the incremental benefit of introducing malaria screening strategies to antenatal care in Africa. Nature Communications 2020; 11(1): 3799.
125. Walker P, Floyd J, Ter Kuile F, Cairns M. Modelling the potential incremental value of intermittent screening and treatment in sub-Saharan Africa. 2015; 2015.
126. Walker P, Ghani AC, ter Kuile FO, Cairns M. Evaluating the cost-effectiveness of intermittent screening and treatment (IST) compared to intermittent preventative therapy (IPTp) during pregnancy in preventing low birth weight: a model-based analysis. 2012; 2012. p. 272-.
127. Webster, J, Mishra, et al. Evaluation of implementation of intermittent screening and treatment for control of malaria in pregnancy in Jharkhand, India. American Journal of Tropical Medicine and Hygiene 2020.
128. Williams J, Njie F, Cairns M, et al. Non-falciparum malaria infections in pregnant women in West Africa. Malaria Journal 2016; 15(1): 53.
129. Williams JE, Cairns M, Njie F, et al. The performance of a rapid diagnostic test in detecting malaria infection in pregnant women and the impact of missed infections. Clinical Infectious Diseases 2015; 62(7): 837-44.
130. Williams JE, Cairns M, Njie F, et al. The Performance of a Rapid Diagnostic Test in Detecting Malaria Infection in Pregnant Women and the Impact of Missed Infections. Clin Infect Dis 2016; 62(7): 837-44.
131. Williams JEO. The prevalence and importance of malaria infections during pregnancy not detected by microscopy or rapid diagnostic testing; 2018.
132. Willilo R, Mandike R, Mugarula F, Kafuko J, Mahdi R, Ekenna U. Monitoring malaria parasitemia prevalence among pregnant women at reproductive and child health clinics in the Lake zone, Tanzania. 2013; 2013.
133. World Health Organisation. Three interlinked patient monitoring systems for HIV care/ART, MCH/PMTCT (including malaria prevention during pregnancy) and TB/HIV: standardized minimum data set and illustrative tools, 2010.
134. World Health Organisation, Malaria Policy Advisory Committee M. Meeting report of the WHO evidence review group on malaria in pregnancy, 2017.
135. Yakoob MY, Lawn JE, Darmstadt GL, Bhutta ZA. Stillbirths: epidemiology, evidence, and priorities for action. Seminars in Perinatology 2010; 34(6): 387-94.
136. Zhou Z, Gutman JR, Mwandama D, et al. Comparative analysis of malaria infections by nested PCR using a pooling strategy on dried blood sports and placental histology in microscopy-negative Malawian women on IPTp. American Society of Tropical Medicine and Hygiene 62nd Annual Meeting 2013; Abstract Book: p. 381.

Supplement 8: Sensitivity analysis of method of assessment for gestational age

A comparison between the Ballard score at delivery[12] and dating ultrasound (at enrolment) showed that the Ballard methods underestimated the mean gestational age at delivery by an average of 2.54 days (95% CI 1.67-3.41, p <0.001) overall. This was 1.64 days (95% CI 0.04-3.25, p=0.04) for women who had any evidence of malaria infection during pregnancy (either patent or subpatent), and 2.66 days (0.43-4.89, p=0.021) in women without any malaria. The corresponding figures were 3.52 days

(1.62-5.41, p<0.001) and 2.78 days (1.28-4.28, p=0.001) for women with patent infections only and for women who had subpatent infections only, respectively (Table S5). The impact of the method of assessment on the mean gestational age estimates at birth between patent and subpatent infection were modest (3.52 vs 2.78 days), and the conclusions of the meta-analyses were the same regardless of whether ultrasound or the Ballard score were used to assess gestational age.

Supplemental references

1. Higgins JPT, Savović J, Page MJ, Elbers RG, Sterne JAC. Chapter 8: Assessing risk of bias in a randomized trial. In: Higgins JPT TJ, Chandler J, Cumpston M, Li T, Page MJ, Welch VA, ed. Cochrane Handbook for Systematic Reviews of Interventions version 61 (updated September 2020) Available from wwwtrainingcochraneorg/handbook: Cochrane; 2020.
2. Villar J, Cheikh Ismail L, Victora CG, et al. International standards for newborn weight, length, and head circumference by gestational age and sex: the Newborn Cross-Sectional Study of the INTERGROWTH-21st Project. *Lancet (London, England)* 2014; **384**(9946): 857-68.
3. Tagbor H, Bruce J, Agbo M, Greenwood B, Chandramohan D. Intermittent screening and treatment versus intermittent preventive treatment of malaria in pregnancy: a randomised controlled non-inferiority trial. *PloS one* 2010; **5**(12): e14425.
4. Desai M, Gutman J, L'Lanziva A, et al. Intermittent screening and treatment or intermittent preventive treatment with dihydroartemisinin-piperaquine versus intermittent preventive treatment with sulfadoxine-pyrimethamine for the control of malaria during pregnancy in western Kenya: an open-label, three-group, randomised controlled superiority trial. *Lancet* 2015.
5. Madanitsa M, Kalilani L, Mwapasa V, et al. Scheduled Intermittent Screening with Rapid Diagnostic Tests and Treatment with Dihydroartemisinin-Piperaquine versus Intermittent Preventive Therapy with Sulfadoxine-Pyrimethamine for Malaria in Pregnancy in Malawi: An Open-Label Randomized Controlled Trial. *PLoS medicine* 2016; **13**(9): e1002124.
6. Tagbor H, Cairns M, Bojang K, et al. A Non-Inferiority, Individually Randomized Trial of Intermittent Screening and Treatment versus Intermittent Preventive Treatment in the Control of Malaria in Pregnancy. *PloS one* 2015; **10**(8): e0132247.
7. Esu E, Berens-Riha N, Pritsch M, Nwachuku N, Loescher T, Meremikwu M. Intermittent screening and treatment with artemether-lumefantrine versus intermittent preventive treatment with sulfadoxine-pyrimethamine for malaria in pregnancy: a facility-based, open-label, non-inferiority trial in Nigeria. *Malaria journal* 2018; **17**(1): 251.
8. Noel-Weiss J, Courant G, Woodend AK. Physiological weight loss in the breastfed neonate: a systematic review. *Open medicine : a peer-reviewed, independent, open-access journal* 2008; **2**(4): e99-e110.
9. Flaherman VJ, Kuzniewicz MW, Li S, Walsh E, McCulloch CE, Newman TB. First-day weight loss predicts eventual weight nadir for breastfeeding newborns. *Archives of disease in childhood Fetal and neonatal edition* 2013; **98**(6): F488-92.
10. Greenwood BM, Greenwood AM, Snow RW, Byass P, Bennett S, Hatib-N'Jie AB. The effects of malaria chemoprophylaxis given by traditional birth attendants on the course and outcome of pregnancy. *Transactions of the Royal Society of Tropical Medicine and Hygiene* 1989; **83**(5): 589-94.
11. D'Alessandro U, Langerock P, Bennett S, Francis N, Cham K, Greenwood BM. The impact of a national impregnated bed net programme on the outcome of pregnancy in primigravidae in The Gambia. *Transactions of the Royal Society of Tropical Medicine and Hygiene* 1996; **90**(5): 487-92.
12. Ballard JL, Khoury JC, Wedig K, Wang L, Eilers-Walsman BL, Lipp R. New Ballard Score, expanded to include extremely premature infants. *J Pediatr* 1991; **119**(3): 417-23.
13. Papageorghiou AT, Kennedy SH, Salomon LJ, et al. The INTERGROWTH-21(st) fetal growth standards: toward the global integration of pregnancy and pediatric care. *Am J Obstet Gynecol* 2018; **218**(2S): S630-S40.

14. Fox GA, Negrete-Yankelevich S, Sosa VJ. Ecological Statistics: Contemporary theory and application. Oxford and New York: Oxford University Press; 2015.
15. Borenstein M, Hedges LS, Higgens JPT, Rothstein HR. Chapter 13: Fixed-Effect Versus Random-Effects Models. Introduction to meta-analysis. Chichester, United Kingdom: John Wiley & Sons, Ltd; 2009.
16. Seide SE, Rover C, Friede T. Likelihood-based random-effects meta-analysis with few studies: empirical and simulation studies. *BMC Med Res Methodol* 2019; **19**(1): 16.
17. Higgins JP, Thompson SG, Deeks JJ, Altman DG. Measuring inconsistency in meta-analyses. *BMJ* 2003; **327**(7414): 557–60.
18. Deeks JJ, Higgins JP, Altman DG. Analysing data and undertaking meta-analyses. In: Higgins JP, Green S, eds. Cochrane handbook for systematic reviews of interventions (version 510): The Cochrane Collaboration; 2011.
19. Cummings P. Methods for estimating adjusted risk ratios. *The Stata Journal* 2009; **9**(2): 175-96.
20. Taylor SM, Madanitsa M, Thwai KL, et al. Minimal Impact by Antenatal Subpatent Plasmodium falciparum Infections on Delivery Outcomes in Malawian Women: A Cohort Study. *The Journal of infectious diseases* 2017; **216**(3): 296-304.
21. [No authors listed]. Committee Opinion No 700: Methods for Estimating the Due Date. *Obstet Gynecol* 2017; **129**(5): e150-e4.
22. Villar J, Papageorghiou AT, Pang R, et al. The likeness of fetal growth and newborn size across non-isolated populations in the INTERGROWTH-21st Project: the Fetal Growth Longitudinal Study and Newborn Cross-Sectional Study. *The Lancet Diabetes & Endocrinology* 2014; **2**(10): 781-92.
23. Schmiegelow C, Scheike T, Oesterholt M, et al. Development of a fetal weight chart using serial trans-abdominal ultrasound in an East African population: a longitudinal observational study. *PloS one* 2012; **7**(9): e44773.
24. Landis SH, Ananth CV, Lokomba V, et al. Ultrasound-derived fetal size nomogram for a sub-Saharan African population: a longitudinal study. *Ultrasound in Obstetrics & Gynecology* 2009; **34**(4): 379-86.

Supplemental figures

Figure S1: Risk of Bias Assessment

Traffic light plot

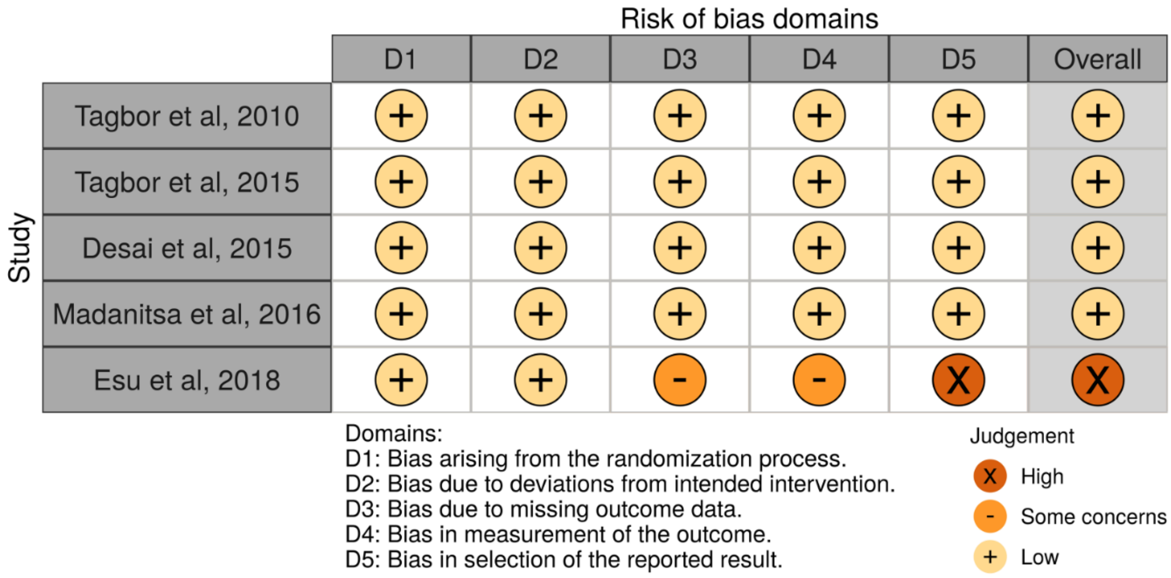

Summary plot

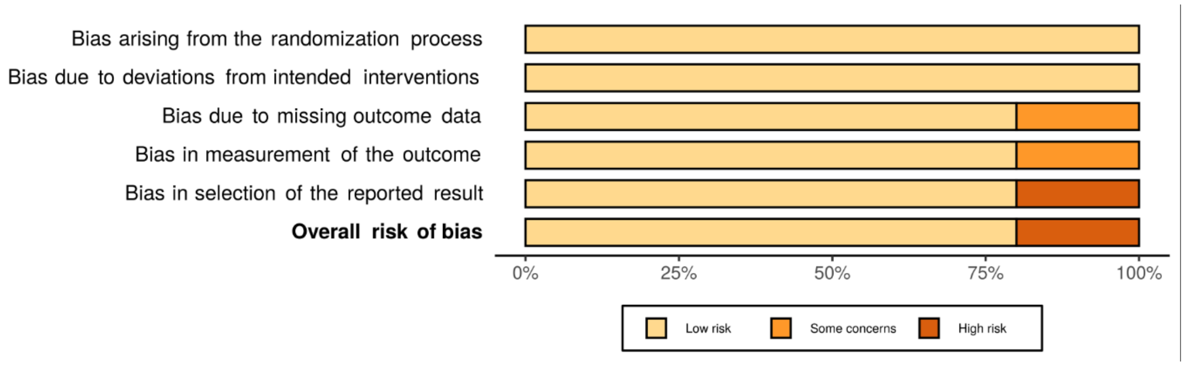

Esu et al., 2018 was scored as ‘Some concern’ for domain 3 (D3) because the primary outcome (haemoglobin concentration in the third trimester) was missing in 48.1% of participants, and the key secondary outcomes, malaria microscopy in the third trimester and birthweight, were missing in 54.7% and 29.2% of participants, respectively. It was also scored as ‘Some concern’ for domain 4 (D4) because only one preterm delivery was recorded, which is much lower than the expected background rate, potentially biasing the effect size towards the null. Domain 5 (D5) was scored as ‘High’ because not all outcomes were reported for the per-protocol or intention to treat analysis and because the p-value of the relative risk for LBW was P=0.7 in the main text, but the effect on the risk difference in LBW is reported as being significant elsewhere (p <0.001) and in the abstract.

The plots were generated using the Risk of Bias visualisation tool obtained from McGuinness, LA, Higgins, JPT. Risk-of-bias Visualization (robvis): An R package and Shiny web app for visualizing risk-of-bias assessments. Res Syn Meth. 2020; 1- 7. https://doi.org/10.1002/jrsm.1411

Supplemental tables

TABLE S1: STUDY CHARACTERISTICS OF INCLUDED TRIALS

Author and Publication Year	Tagbor et al., 2010	Tagbor et al., 2015	Desai et al., 2015	Madanitsa et al., 2016	Esu et al., 2018
**Country**	Ghana	The Gambia, Mali, Burkina Faso and Ghana	Kenya	Malawi	Nigeria
**Recruitment**	March 2007 – September 2007	May 2010 – October 2011	August 2012 – June 2014	July 2011 – March 2013	October 2013 – November 2014*
**Design**	Non-inferiority	Non-inferiority	Superiority	Superiority	Non-inferiority
**Number of sites**	1	6†	4	3	1
**SP resistance ‡ (Ala437Gly | Lys540Glu | Ala581Gly)**	No molecular data available, but generally low SP resistance	BF: 75.3 |0.0|0.0<br>Ghana: 77.4 |0.0|0.0<br>Gambia: 9.1 |0.0|0.0<br>Mali: 21.4 |0.37|0[10]	93.0 |95.6|5.7[16]	94.4 |99.6|1.5[16]	No molecular data available, but generally low SP resistance
**Gravidity groups**	All	G1/G2 only	All	All; enrolment stratified by G1/2 and G3+	All
**Gestational age at enrolment**	16-24	16-30	16-32	16-28	16-24
**Method of gestational age assessment**	Ballard	Ballard	Ballard	Ultrasound	
**Intervention frequency**	IPTp or IST up to 3 times depending on GA at enrolment	IPTp up to 2 times in The Gambia, Mali, Burkina Faso and up to 3 times in Ghana as per national policy. ISTp up to 3 times depending on GA at enrolment	IPTp or ISTp up to 4 times depending on GA at enrolment	IPTp or ISTp up to 4 times depending on GA at enrolment	IPTp-SP or ISTp administered up to 3 times (at enrolment and 24 and 32 weeks GA)
**Drug used for ISTp**	SP or AQ+AS§	AL	DP	DP	AL
**RDT used for ISTp**	DiaMed OptiMAL-IT, pLDH based RDT (Cressier, Switzerland)	First Response Malaria pLDH/HRP2 Combo Test (Premier Medical Corporation Ltd., Mumbai, India)	First Response Malaria pLDH/HRP2 Combo Test (Premier Medical Corporation Ltd., Mumbai, India)	First Response Malaria pLDH/HRP2 Combo Test (Premier Medical Corporation Ltd., Mumbai, India)	SD Bioline malaria antigen HRP2/pLDH (Pan) RDT
**Primary outcome**	Third trimester anaemia	Low birth weight	Malaria infection at delivery	Muligravidae: Malaria infection at delivery<br>Paucigravidae: SGA/ LBW/ preterm composite	Third trimester haemoglobin concentration
**Proportion contributing to study primary outcome (ITT)**	80.0%	86.9%	88.5%	SGA/LBW/preterm: 90.9%<br>Malaria infection:88.3%	52.1%

**Baseline malaria prevalence (%) by microscopy and seasonality**	17.4%, perennial transmission with marked seasonality	30.8%, all sites had marked seasonality	17.3%, perennial transmission	15.9%, perennial transmission with marked seasonality	7.0%, perennial transmission with marked seasonality
**Folate dose (mg/day)**	4	0.4	0.4	0.4	4
**Number of women that received at least one course of artemether-lumefantrine or amodiaquine-artesunate**	ISTp: 252 (22.8%)<br>IPTP: 2 (0.2%)	ISTp: 1334 (50.4%)<br>IPTP: 18 (0.7%)	ISTp: 61 (11.9%)<br>IPTP: 45 (8.9%)	ISTp: 0 (0%)<br>IPTP: 144 (15.6%)	N/A
**Number of courses of artemether-lumefantrine received for ISTp¶**	252, 1.2 (0.5) [1-4]	1334, 1.3 (0.6) [1-3]	195, 1.8 (0.8) [1-4]	473, 1.3 (0.5) [1-3]	N/A
**Number of SP courses received for IPTp¶**	1098, 2.3 (1.0) [1-6]	2634, 2.1 (0.5) [1-4]	506, 4.4 (1.2) [2-7]	916, 3.3 (0.9) [1-4]	N/A

Values are mean (SD) or percentages unless otherwise indicated.
GA=gestational age. ISTp=intermittent screening and treatment in pregnancy. IPTp=intermittent preventive treatment in pregnancy. AQ+AS=amodiaquine+artesunate. AL=artesunate-lumefantrine. DP=dihydroartemisinin-piperaquine. SP=sulfadoxine-pyrimethamine. BF Burkina-Faso. EIR=Entomological inoculation rate (infectious bites/person/year). ITN=insecticide treated nets. SES=Socioeconomic status. ITT=Intention to treat population.

* Esu: Dates reported correspond to recruitment and follow-up.
† One site in Burkina Faso, the Gambia, and Ghana, and three sites in Mali. All sites contributed to efficacy analysis comparing ISTp vs IPTp (except for outcomes requiring PCR data). Only Ghana had serial PCR data and contributed to the subpatent IPD meta-analysis.
‡ Prevalence of Ala437Gly, Lys540Glu, and Ala581Gly substitutions in the parasite dihydropteroate synthase gene
§ Only data from the ISTp arm with amodiaquine+artesunate was used in this analysis
¶ Values are N, mean (SD) [range].

Table S2: Baseline characteristics of participants

	Tagbor et al., 2010	Tagbor et al., 2015	Desai et al., 2015	Madanitsa et al., 2016	Esu et al., 2018
**N**	**2205**	**5292**	**1021**	**1844**	**459**
**Maternal characteristics**					
Maternal age (years)	26.5 (6.0)	20.4 (3.3)	23.4 (5.9)	22.5 (5.1)	28.2 (5.0)
Used a bednet previous night	1133 (71.5%)	3063 (58.8%)	730 (71.5%)	350 (19.0%)	67 (14.6%)
Schooling level*					
Low	438 (27.4%)	4537 (86.4%)	235 (23.1%)	565 (30.7%)	13 (2.8%)
Medium	1125 (70.4%)	635 (12.1%)	452 (44.4%)	980 (53.3%)	195 (42.5%)
High	34 (2.1%)	78 (1.5%)	330 (32.4%)	295 (16.0%)	251 (54.7%)
SES index score (median, range)	0.1 (-7.9; 4.1)	0.4 (-6.0; 4.9)	-0.2 (-8.3; 19.6)	-1.0 (-1.8; 14.7)	N/A
Pregnancy number (gravidity)					
First	505 (22.9%)	2904 (55.1%)	353 (34.6%)	627 (34.1%)	216 (47.0%)
Second	486 (22.1%)	2363 (44.9%)	204 (20.0%)	513 (27.9%)	137 (29.9%)
Third or higher	1210 (55.0%)	0 (0.0%)	464 (45.4%)	704 (38.2%)	106 (23.1%)
Gestational age (weeks)	19.8 (2.7)	20.5 (3.3)	22.9 (4.8)	20.9 (3.1)	N/A
Weight (kg)	59.5 (10.7)	N/A	61.3 (8.7)	55.1 (7.4)	N/A
Height (cm)	155.3 (8.2)	N/A	164.2 (6.9)	154.0 (5.0)	N/A
BMI	24.6 (4.0)	N/A	22.7 (3.0)	23.2 (3.0)	N/A
**Laboratory findings**					
Haemoglobin (g/dL)	10.7 (1.4)	10.4 (2.3)	10.5 (1.6)	11.0 (1.5)	11.5 (1.4)
Malaria infection					
RDT†	189 (15.2%)	1045 (40.2%)	111 (22.9%)	385 (38.4%)	26 (11.3%)‡
Microscopy	368 (17.4%)	1595 (30.8%)	167 (17.3%)	291 (15.9%)	32 (7.0%)
PCR		1216 (40.8%)	330 (34.1%)	788 (43.6%)	29 (6.3%)
Subpatent		331 (9.2%)	171 (20.0%)	360 (27.4%)	

Values are mean (SD) or percentages unless otherwise indicated.

SES=Socioeconomic status. N/A=Not available. RDT=Rapid diagnostic test for malaria. PCR=Polymerase chain reaction. BMI=Body mass index.

* Schooling: Low: no schooling or primary school not completed, Medium: Primary school completed, High: Junior high school completed, Highest: Senior High school or academy completed. For Esu et al., Low corresponds to primary school attainment, medium to secondary school, and high to tertiary school.

† RDT were only conducted in symptomatic women in the IPTp arms, but among all women in the ISTp arm. ‡ The RDT data reported for Esu represent the ISTp-AL arm only (n=230).

Table S3: Fixed versus random-effects models of the comparison between ISTp-ACT vs IPTp-SP

Outcome	# studies	Fixed-effect* RR (95%CI), P-value	Random-effects* RR (95%CI), P-value	I^2 (%)
**Adverse pregnancy outcome (co-primary outcome)†**				
Low resistance	2	1.01 (0.97;1.06), 0.637	1.01 (0.97;1.06), 0.637	0.0
High resistance	2	0.94 (0.83;1.07), 0.341	1.07 (0.71;1.61), 0.760	81.3
Overall	4	1.00 (0.96;1.05), 0.916	1.00 (0.92;1.09), 0.968	54.5
**Any Plasmodium infection at delivery (placental or peripheral) (co-primary outcome)‡**				
Low resistance	1	1.00 (0.90;1.10), 0.960	1.00 (0.90;1.10), 0.960	0.0
High resistance	2	1.20 (1.06;1.35), 0.003	1.20 (1.06;1.35), 0.003	0.0
Overall	3	1.08 (1.00;1.16), 0.063	1.09 (0.94;1.28), 0.253	67.0
**Any subpatent Plasmodium infection at delivery (placental or peripheral)§**				
Low resistance	1	1.08 (0.61;1.89), 0.795	1.08 (0.61;1.89), 0.795	0.0
High resistance	2	1.17 (0.99;1.39), 0.066	1.05 (0.68;1.61), 0.831	65.7
Overall	3	1.17 (0.99;1.37), 0.066	1.10 (0.84;1.43), 0.488	33.3
**Any patent Plasmodium infection at delivery (placental or peripheral)§**				
Low resistance	1	0.81 (0.55;1.19), 0.283	0.81 (0.55;1.19), 0.283	0.0
High resistance	2	1.28 (1.00;1.64), 0.048	1.28 (1.00;1.64), 0.048	0.0
Overall	3	1.12 (0.91;1.38), 0.278	1.10 (0.81;1.49), 0.533	51.4
**Any placental Plasmodium infection (patent or subpatent)¶**				
Low resistance	1	0.98 (0.88;1.09), 0.764	0.98 (0.88;1.09), 0.764	0.0
High resistance	2	1.18 (1.02;1.37), 0.022	1.18 (1.02;1.37), 0.022	0.0
Overall	3	1.05 (0.96;1.14), 0.268	1.08 (0.93;1.25), 0.319	53.7
**Subpatent placental Plasmodium infection||**				
Low resistance	0	---	---	
High resistance	2	1.12 (0.90;1.41), 0.311	1.11 (0.85;1.44), 0.441	11.3
Overall	2	1.12 (0.90;1.41), 0.311	1.11 (0.85;1.44), 0.441	11.3
**Patent placental Plasmodium infection||**				
Low resistance	0	---	---	
High resistance	2	1.30 (1.00;1.70), 0.051	1.30 (1.00;1.70), 0.051	0.0
Overall	2	1.30 (1.00;1.70), 0.051	1.30 (1.00;1.70), 0.051	0.0
**Any maternal peripheral blood Plasmodium infection at delivery (patent or subpatent)****				
Low resistance	2	1.09 (0.95;1.26), 0.227	1.09 (0.95;1.26), 0.227	0.0
High resistance	2	1.30 (1.11;1.52), 0.001	1.30 (1.11;1.52), 0.001	0.0
Overall	4	1.18 (1.06;1.31), 0.002	1.18 (1.06;1.31), 0.002	0.0
**Subpatent maternal peripheral blood Plasmodium infection at delivery††**				
Low resistance	1	1.12 (0.63;1.97), 0.707	1.12 (0.63;1.97), 0.707	0.0
High resistance	2	1.34 (1.06;1.69), 0.013	1.34 (1.06;1.69), 0.013	0.0
Overall	3	1.31 (1.05;1.62), 0.015	1.31 (1.05;1.62), 0.015	0.0
**Patent maternal peripheral blood Plasmodium infection at delivery††**				
Low resistance	1	0.83 (0.57;1.23), 0.358	0.83 (0.57;1.23), 0.358	0.0
High resistance	2	1.15 (0.86;1.54), 0.354	1.15 (0.86;1.54), 0.354	0.0
Overall		1.02 (0.81;1.29), 0.853	1.02 (0.81;1.29), 0.853	0.0
**Any anaemia in the third trimester/delivery (Hb<11 g/dL)‡‡**				
Low resistance	3	1.02 (0.96;1.08), 0.510	1.02 (0.96;1.08), 0.510	0.0
High resistance	2	0.91 (0.81;1.03), 0.133	0.92 (0.78;1.09), 0.315	47.4
Overall	5	1.00 (0.95;1.05), 0.962	0.98 (0.91;1.06), 0.606	38.4

Outcome	# studies	Fixed-effect* RR (95%CI), P-value	Random-effects* RR (95%CI), P-value	I^2 (%)
**Moderate-to-severe anaemia in the third trimester/delivery (Hb<9 g/dL)‡‡**				
Low resistance	2	1.18 (0.96;1.46), 0.108	1.18 (0.96;1.46), 0.108	0.0
High resistance	2	1.02 (0.68;1.53), 0.918	1.02 (0.68;1.53), 0.918	0.0
Overall	4	1.15 (0.96;1.38), 0.139	1.15 (0.96;1.38), 0.139	0.0
**Clinical malaria during pregnancy§§**				
Low resistance	2	1.44 (1.13;1.85), 0.004	1.44 (1.13;1.85), 0.004	0.0
High resistance	2	1.08 (0.88;1.32), 0.453	1.09 (0.84;1.42), 0.499	37.2
Overall	4	1.21 (1.04;1.42), 0.016	1.23 (1.00;1.51), 0.054	37.2
**Any Plasmodium infection during pregnancy (patent or subpatent)¶¶**				
Low resistance	3	1.32 (1.22;1.43), <.001	1.32 (1.22;1.43), <.001	0.0
High resistance	2	1.10 (1.02;1.19), 0.017	1.14 (0.95;1.36), 0.166	75.8
Overall	5	1.20 (1.14;1.28), <.001	1.21 (1.06;1.38), 0.005	74.0
**Patent Plasmodium Infection during pregnancy||||**				
Low resistance	3	1.47 (1.34;1.60), <.001	1.39 (1.14;1.70), 0.001	56.3
High resistance	2	1.21 (1.05;1.41), 0.011	1.22 (0.94;1.59), 0.133	67.8
Overall	5	1.40 (1.29;1.50), <.001	1.31 (1.11;1.55), 0.001	67.7
**Subpatent Plasmodium infection during pregnancy||||**				
Low resistance	1	0.87 (0.63;1.21), 0.409	0.87 (0.63;1.21), 0.409	0.0
High resistance	2	1.01 (0.90;1.13), 0.898	1.01 (0.90;1.13), 0.898	0.0
Overall	3	0.99 (0.89;1.10), 0.885	0.99 (0.89;1.10), 0.885	0.0
**Low birth weight (<2,500g)**				
Low resistance	3	1.04 (0.93;1.17), 0.489	1.04 (0.93;1.17), 0.489	0.0
High resistance	2	1.28 (0.99;1.64), 0.055	1.28 (0.99;1.64), 0.055	0.0
Overall	5	1.08 (0.97;1.20), 0.154	1.09 (0.97;1.23), 0.146	7.5
**Preterm birth (<37 weeks)**				
Low resistance	3	0.99 (0.92;1.07), 0.818	0.99 (0.92;1.07), 0.818	0.0
High resistance	2	1.04 (0.88;1.24), 0.620	1.18 (0.74;1.88), 0.481	57.3
Overall	5	1.00 (0.93;1.07), 0.999	1.00 (0.93;1.07), 0.999	0.0
**Small-for-gestational-age**				
Low resistance	2	1.03 (0.95;1.11), 0.528	1.03 (0.95;1.11), 0.528	0.0
High resistance	2	0.83 (0.67;1.04), 0.111	0.94 (0.47;1.89), 0.856	88.1
Overall	4	1.00 (0.93;1.08), 0.962	0.99 (0.80;1.21), 0.892	74.9
**Spontaneous miscarriage (<28 weeks gestation)**				
Low resistance	3	0.80 (0.46;1.39), 0.424	0.80 (0.46;1.39), 0.424	0.0
High resistance	2	2.02 (0.60;6.77), 0.257	2.02 (0.60;6.77), 0.257	0.0
Overall	5	0.94 (0.57;1.55), 0.797	0.94 (0.57;1.55), 0.797	0.0
**Stillbirth (>=28 weeks gestation)**				
Low resistance	3	1.15 (0.87;1.51), 0.331	1.15 (0.87;1.51), 0.331	0.0
High resistance	2	1.06 (0.60;1.88), 0.834	1.10 (0.35;3.45), 0.872	74.8
Overall	5	1.13 (0.88;1.45), 0.334	1.14 (0.84;1.53), 0.396	14.1
**Fetal loss (spontaneous abortion or stillbirth)**				
Low resistance	3	1.08 (0.83;1.39), 0.573	1.08 (0.83;1.39), 0.573	0.0
High resistance	2	1.19 (0.71;2.00), 0.505	1.16 (0.37;3.63), 0.793	79.4
Overall	5	1.10 (0.87;1.38), 0.424	1.09 (0.79;1.51), 0.586	34.8

Outcome	# studies	Fixed-effect* RR (95%CI), P-value	Random-effects* RR (95%CI), P-value	I^2 (%)
**Perinatal death**				
Low resistance	2	1.07 (0.84;1.36), 0.589	1.07 (0.84;1.36), 0.589	0.0
High resistance	2	0.98 (0.63;1.50), 0.914	0.98 (0.37;2.62), 0.971	80.7
Overall	4	1.05 (0.85;1.29), 0.675	1.05 (0.74;1.50), 0.777	46.4
**Neonatal death (<28 days)**				
Low resistance	1	1.03 (0.69;1.53), 0.887	1.03 (0.69;1.53), 0.887	0.0
High resistance	2	0.93 (0.48;1.79), 0.824	0.90 (0.31;2.59), 0.846	61.2
Overall	3	1.00 (0.71;1.40), 0.994	0.99 (0.63;1.54), 0.949	24.5
**Infant mortality by end of follow-up(6-8 weeks of age)**				
Low resistance	1	1.07 (0.73;1.56), 0.743	1.07 (0.73;1.56), 0.743	0.0
High resistance	2	0.91 (0.49;1.68), 0.752	0.85 (0.29;2.52), 0.767	67.1
Overall	3	1.02 (0.74;1.41), 0.910	0.98 (0.60;1.60), 0.929	38.1
**Fetal loss or infant death by 6-8 weeks**				
Low resistance	1	1.10 (0.88;1.39), 0.404	1.10 (0.88;1.39), 0.404	0.0
High resistance	2	1.07 (0.72;1.58), 0.742	1.02 (0.34;3.06), 0.976	87.3
Overall	3	1.09 (0.90;1.34), 0.376	1.06 (0.64;1.75), 0.821	74.7
**Congenital anomalies**				
Low resistance	2	1.28 (0.68;2.41), 0.438	1.28 (0.68;2.41), 0.438	0.0
High resistance	2	0.81 (0.48;1.34), 0.409	0.81 (0.48;1.34), 0.409	0.0
Overall	4	0.97 (0.65;1.44), 0.879	0.97 (0.65;1.44), 0.879	0.0
**Maternal Hb (g/dL)(last visit)**				
Low resistance	3	-0.01 (-0.08;0.06), 0.822	0.01 (-0.10;0.11), 0.905	42.5
High resistance	2	0.06 (-0.05;0.17), 0.275	0.04 (-0.14;0.21), 0.686	50.7
Overall	4	0.01 (-0.05;0.07), 0.688	0.02 (-0.06;0.10), 0.635	39.3
**Fetal haemoglobin (g/dL) (cord blood)**				
Low resistance	0	---	---	
High resistance	2	-0.08 (-0.27;0.11), 0.411	-0.11 (-0.41;0.19), 0.464	56.1
Overall	2	-0.08 (-0.27;0.11), 0.411	-0.11 (-0.41;0.19), 0.464	56.1
**Birth weight (grams)**				
Low resistance	3	-24.7 (-46.2;-3.2), 0.024	-24.7 (-46.2;-3.2), 0.024	0.0
High resistance	2	-36.9 (-71.5;-2.3), 0.037	-36.9 (-71.5;-2.3), 0.037	0.0
Overall	5	-28.1 (-46.4;-9.8), 0.003	-28.1 (-46.4;-9.8), 0.003	0.0
**Birthweight for gestational age (Z-score)**				
Low resistance	2	-0.05 (-0.15;0.05), 0.311	-0.05 (-0.15;0.05), 0.311	0.0
High resistance	2	-0.02 (-0.11;0.07), 0.628	-0.02 (-0.11;0.07), 0.628	0.0
Overall	4	-0.03 (-0.10;0.03), 0.303	-0.03 (-0.10;0.03), 0.303	0.0
**Gestational age at birth (weeks)**				
Low resistance	2	-0.03 (-0.20;0.14), 0.708	-0.03 (-0.20;0.14), 0.708	0.0
High resistance	2	-0.17 (-0.33;-0.01), 0.04	-0.17 (-0.33;-0.01), 0.04	0.0
Overall	4	-0.10 (-0.22;0.01), 0.082	-0.10 (-0.22;0.01), 0.082	0.0
**Asexual parasite density (log) (delivery)**				
Low resistance	2	1.11 (0.81;1.52), 0.505	1.11 (0.81;1.52), 0.505	0.0
High resistance	2	1.78 (0.70;4.49), 0.224	1.78 (0.70;4.49), 0.224	0.0
Overall	4	1.17 (0.87;1.57), 0.307	1.17 (0.87;1.57), 0.307	0.0

RR=relative risk, CI=confidence interval

* Fixed-effect using inverse-variance estimation method, Random-effects using the DerSimonian–Laird method.

Outcome	# studies	Fixed-effect* RR (95%CI), P-value	Random-effects* RR (95%CI), P-value	I^2 (%)

† Adverse live-birth (co-primary outcome) defined as the composite of low birth weight (<2500 grams), small-for-gestational-age (SGA, <10th percentile relative to INTERGROWTH-21st gender-specific chart), or and preterm delivery (<37 weeks gestation).
‡ Any malaria at delivery (co-primary outcome) defined as any maternal plasmodium infection detected in peripheral or placental blood by any diagnostic method (PCR, microscopy, RDT or histopathology (acute and/or chronic infections)).
§Any patent *Plasmodium* infection in peripheral or placental blood detected by PCR or histopathology (acute and/or chronic infections) and positive by microscopy or RDT. Any subpatent infection at delivery is defined as a microscopy and RDT negative infection detected by PCR or histopathology.
¶Any placental malaria infection detected in the placental blood by any diagnostic method (PCR, microscopy, RDT or histopathology (acute and/or chronic infections)).
||Patent placental malaria infection defined as any infection in the placental blood detected by PCR or histopathology positive by RDT or microscopy. Subpatent placental malaria infection is defined as microscopy and RDT negative infections detected by PCR or histopathology (acute and/or chronic infections).
**Any maternal plasmodium infection detected in peripheral blood by PCR, microscopy, or RDT.
††Patent maternal plasmodium infection in peripheral blood detected by PCR and by microscopy or RDT. Subpatent maternal plasmodium infection detected in peripheral blood by PCR, but not by microscopy or RDT.
‡‡Maternal anaemia (Hb <11 g/dL) and moderate to severe anaemia (Hb <9 g/dL) at delivery or otherwise in the third trimester if values at delivery were not available.
§§Clinical malaria, defined as documented fever or recent history of fever in the presence of microscopy or RDT confirmed malaria infection.
¶¶Any maternal peripheral blood *Plasmodium* infection during pregnancy, detected by microscopy or RDT, or PCR.
||||Patent maternal peripheral blood *Plasmodium* infection during pregnancy detected by PCR and by microscopy or RDT. Subpatent Plasmodium infection during pregnancy, defined as PCR positive but microscopy and RDT negative infections.

TABLE S4: CONTINUOUS OUTCOMES COMPARING ISTP TO IPTP

Outcome	Study	Number of women, mean (SD) IST	IPT	Unadjusted mean difference (95% CI), p-value*	I^2 (%)	Adjusted mean Difference (95% CI), p-value
**Maternal Hb (g/dL) (delivery or last visit in 3rd trimester)**						
Low resistance	Tagbor, 2010	888, 11.0 (1.2)	881, 11.0 (1.3)	-0.03 (-0.14;0.09), 0.666		-0.02 (-0.15;0.11), 0.816
	Tagbor, 2015	1621, 10.9 (1.4)	1588, 11.0 (1.4)	-0.02 (-0.12;0.07), 0.654		-0.00 (-0.09;0.09), 0.976
	Esu, 2018	124, 11.7 (1.2)	115, 11.4 (1.4)	0.30 (-0.03;0.63), 0.076		
	Subgroup, IV (I^2=42.5%, p=0.175)			-0.01 (-0.08;0.06), 0.822	42.5	-0.01 (-0.08;0.07), 0.875
High resistance	Desai, 2015	307, 11.1 (1.3)	294, 11.1 (1.5)	-0.08 (-0.31;0.14), 0.480		-0.06 (-0.27;0.16), 0.614
	Madanitsa, 2016	670, 11.7 (1.2)	665, 11.6 (1.2)	0.11 (-0.02;0.23), 0.099		0.11 (-0.02;0.23), 0.091
	Subgroup, IV (I^2=50.7%, p=0.155)			0.06 (-0.05;0.17), 0.275	50.7	0.07 (-0.04;0.17), 0.218
Overall	Heterogeneity between subgroups: p=0.299			0.01 (-0.05;0.07), 0.688	39.3	0.02 (-0.04;0.08), 0.565
**Fetal haemoglobin (g/dL) (cord blood)**						
Low resistance	Tagbor, 2010	0, . (.)	0, . (.)	. (.;.), .		. (.;.), .
	Tagbor, 2015	0, . (.)	0, . (.)	. (.;.), .		. (.;.), .
	Subgroup, IV (I^2=0.0%, p=.)			. (.;.), .		. (.;.), .
High resistance	Desai, 2015	399, 14.1 (2.4)	403, 14.4 (2.6)	-0.30 (-0.63;0.04), 0.087		-0.29 (-0.63;0.05), 0.089
	Madanitsa, 2016	769, 14.9 (2.4)	761, 14.9 (2.2)	0.02 (-0.21;0.24), 0.884		0.02 (-0.21;0.24), 0.885
	Subgroup, IV (I^2=56.1%, p=0.131)			-0.08 (-0.27;0.11), 0.411	56.1	-0.08 (-0.26;0.11), 0.415
Overall	Heterogeneity between subgroups: p cannot be computed			-0.08 (-0.27;0.11), 0.411	56.1	-0.08 (-0.26;0.11), 0.415
**Birth weight (grams)**						
Low resistance	Tagbor, 2010	858, 2951.4 (475.1)	850, 2987.0 (485.5)	-28 (-72;17), 0.227		-18 (-72;35), 0.497
	Tagbor, 2015	2282, 2830.3 (450.6)	2314, 2857.1 (436.4)	-23 (-48;2), 0.071		-23 (-48;2), 0.073
	Esu, 2018	167, 3170 (0.53)	158, 3210 (0.53)	-40 (-156;76), 0.498		
	Subgroup, IV (I^2=0.0%, p=0.951)			-25 (-46;-3), 0.024	0.0	-35 (-69;-0), 0.048
High resistance	Desai, 2015	412, 3226.5 (493.8)	412, 3275.7 (467.9)	-57 (-120;7), 0.082		-51 (-114;12), 0.113
	Madanitsa, 2016	830, 2919.2 (439.1)	826, 2947.2 (447.6)	-29 (-70;13), 0.174		-28 (-69;13), 0.184
	Subgroup, IV (I^2=0.0%, p=0.470)			-37 (-72;-2), 0.037	0.0	-22 (-45;1), 0.056
Overall	Heterogeneity between subgroups: p=0.558			-28 (-46;-10), 0.003	0.0	-26 (-45;-7), 0.007

**Birthweight for gestational age (Z-score)**						
Low resistance	Tagbor, 2010	759, 0.15 (1.83)	763, 0.22 (1.78)	-0.06 (-0.24;0.13), 0.546		-0.04 (-0.27;0.18), 0.705
	Tagbor, 2015	2012, 0.04 (2.00)	2009, 0.10 (2.02)	-0.05 (-0.17;0.07), 0.415		-0.05 (-0.18;0.07), 0.382
	Subgroup, IV (I^2=0.0%, p=0.960)			-0.05 (-0.15;0.05), 0.311	0.0	-0.01 (-0.10;0.07), 0.771
High resistance	Desai, 2015	410, 0.13 (1.05)	412, 0.18 (1.00)	-0.07 (-0.20;0.07), 0.321		-0.06 (-0.19;0.08), 0.413
	Madanitsa, 2016	815, 0.05 (1.18)	814, 0.04 (1.24)	0.01 (-0.10;0.13), 0.829		0.02 (-0.10;0.13), 0.749
	Subgroup, IV (I^2=0.0%, p=0.371)			-0.02 (-0.11;0.07), 0.628	0.0	-0.05 (-0.16;0.06), 0.343
Overall	Heterogeneity between subgroups: p=0.656			-0.03 (-0.10;0.03), 0.303	0.0	-0.03 (-0.10;0.04), 0.411
**Gestational age at birth (weeks)**						
Low resistance	Tagbor, 2010	909, 38.7 (3.5)	924, 38.6 (3.4)	0.08 (-0.23;0.40), 0.617		-0.01 (-0.39;0.36), 0.953
	Tagbor, 2015	2466, 38.3 (3.7)	2463, 38.4 (3.8)	-0.08 (-0.28;0.12), 0.443		-0.08 (-0.28;0.12), 0.445
	Subgroup, IV (I^2=0.0%, p=0.404)			-0.03 (-0.20;0.14), 0.708	0.0	-0.06 (-0.24;0.11), 0.484
High resistance	Desai, 2015	450, 39.1 (1.8)	451, 39.1 (1.8)	-0.10 (-0.33;0.13), 0.386		-0.11 (-0.34;0.12), 0.352
	Madanitsa, 2016	871, 38.0 (2.4)	864, 38.2 (2.3)	-0.23 (-0.45;-0.00), 0.04		-0.23 (-0.45;-0.01), 0.04
	Subgroup, IV (I^2=0.0%, p=0.451)			-0.17 (-0.33;-0.01), 0.04	0.0	-0.17 (-0.33;-0.01), 0.03
Overall	Heterogeneity between subgroups: p=0.262			-0.10 (-0.22;0.01), 0.082	0.0	-0.12 (-0.24;-0.01), 0.04
**Asexual parasite density maternal blood at delivery (geometric mean, 95% CI)**						
Low resistance	Tagbor, 2010	108, 341 (235;493)	85, 365 (240;555)	0.99 (0.57;1.70), 0.958		0.80 (0.44;1.45), 0.459
	Tagbor, 2015	207, 3467 (2,600;4,623)	186, 2,699 (2,074;3,511)	1.18 (0.80;1.74), 0.395		1.17 (0.80;1.73), 0.417
	Subgroup, IV (I^2=0.0%, p = 0.594)			1.11 (0.81;1.52), 0.505	0.0	1.05 (0.76;1.45), 0.779
High resistance	Desai, 2015	57, 493 (228;1,066)	45, 427 (191;957)	1.89 (0.63;5.64), 0.254		1.82 (0.61;5.42), 0.284
	Madanitsa, 2016	16, 3,861 (1,039;14,345)	18, 2,984 (992;8,979)	1.52 (0.27;8.71), 0.637		0.85 (0.18;3.92), 0.836
	Subgroup, IV (I^2=0.0%, p = 0.836)			1.78 (0.70;4.49), 0.224	0.0	1.41 (0.58;3.42), 0.452
Overall	Heterogeneity between subgroups: p=0.348			1.17 (0.87;1.57), 0.307	0.0	1.08 (0.80;1.47), 0.601

ISTp=intermittent screening and treatment in pregnancy. IPTp=intermittent preventive treatment in pregnancy. I^2=I-squared measure for heterogeneity, based on unadjusted values. I-V=inverse variance method for fixed-effect. Heterogeneity between subgroups represents the difference between the effect in low and high SP resistance areas. When there is considerable heterogeneity observed (I^2 >=75%) in one or more subgroups the tests for heterogeneity between subgroups are likely to be invalid and should be interpreted with caution. Values are mean (SD) unless indicated otherwise. The mean difference for asexual parasite densities at delivery represents the geometric mean ratio.

*The unadjusted mean difference reflects models that include the stratification factor gravidity (primigravidae vs secundigravidae vs multigravidae), which was used in some of the source studies, as well as site. Adjusted models also include anaemia at enrolment (haemoglobin < 11 g/dL), gestational age (binary, study-specific median), and maternal ITN use at enrolment

TABLE S5: COMPARISON OF GESTATIONAL AGE ASSESSMENT BY ULTRASOUND VS BALLARD

	N	Gestational age in days		Mean difference (95% CI) between Ultrasound and Ballard (days)	p-value
		Ultrasound	Ballard		
Overall	921	268.3	265.7	2.5 (1.7-3.4)	<0.001
No malaria	137	267.8	265.2	2.7 (0.4-4.9)	0.02
Patent only	193	269.8	266.3	3.5 (1.6-5.4)	0.001
Subpatent only	288	268.9	266.1	2.8 (1.3-4.3)	0.001
Any malaria	303	266.9	265.3	1.6 (0.0-3.2)	0.04
